# The *DAO* Gene Is Associated with Schizophrenia and Interacts with Other Genes in the Taiwan Han Chinese Population

**DOI:** 10.1371/journal.pone.0060099

**Published:** 2013-03-28

**Authors:** Hsin-Chou Yang, Chih-Min Liu, Yu-Li Liu, Chia-Wei Chen, Chien Ching Chang, Cathy S. J. Fann, Jen-Jie Chiou, Ueng-Cheng Yang, Chun-Houh Chen, Stephen V. Faraone, Ming T. Tsuang, Hai-Gwo Hwu

**Affiliations:** 1 Institute of Statistical Science, Academia Sinica, Taipei, Taiwan; 2 Department of Psychiatry, National Taiwan University Hospital and College of Medicine, National Taiwan University, Taipei, Taiwan; 3 Division of Mental Health and Substance Abuse Research, National Health Research Institutes, Taipei, Taiwan; 4 Institute of Biomedical Sciences, Academia Sinica, Taipei, Taiwan; 5 Institute of Biomedical Informatics, National Yang-Ming University, Taipei, Taiwan; 6 Medical Genetics Research Center and Departments of Psychiatry and Neuroscience and Physiology, SUNY Upstate Medical University, Syracuse, New York, United States of America; 7 Harvard Institute of Psychiatric Epidemiology and Genetics, and Departments of Epidemiology and Psychiatry, Harvard University, Boston, Massachusetts, United States of America; 8 Institute of Epidemiology, College of Public Health, National Taiwan University, Taipei, Taiwan; 9 Department of Psychology, College of Science, National Taiwan University, Taipei, Taiwan; 10 Neurobiology and Cognitive Science Center, National Taiwan University, Taipei, Taiwan; 11 Institute of Behavioral Genomics, University of California San Diego, La Jolla, California, United States of America; National Taiwan University, Taiwan

## Abstract

**Background:**

Schizophrenia is a highly heritable disease with a polygenic mode of inheritance. Many studies have contributed to our understanding of the genetic underpinnings of schizophrenia, but little is known about how interactions among genes affect the risk of schizophrenia. This study aimed to assess the associations and interactions among genes that confer vulnerability to schizophrenia and to examine the moderating effect of neuropsychological impairment.

**Methods:**

We analyzed 99 SNPs from 10 candidate genes in 1,512 subject samples. The permutation-based single-locus, multi-locus association tests, and a gene-based multifactorial dimension reduction procedure were used to examine genetic associations and interactions to schizophrenia.

**Results:**

We found that no single SNP was significantly associated with schizophrenia. However, a risk haplotype, namely *A*-*T*-*C* of the SNP triplet rsDAO7-rsDAO8-rsDAO13 of the *DAO* gene, was strongly associated with schizophrenia. Interaction analyses identified multiple between-gene and within-gene interactions. Between-gene interactions including DAO*DISC1 **,** DAO*NRG1 and DAO*RASD2 and a within-gene interaction for *CACNG2* were found among schizophrenia subjects with severe sustained attention deficits, suggesting a modifying effect of impaired neuropsychological functioning. Other interactions such as the within-gene interaction of *DAO* and the between-gene interaction of *DAO* and *PTK2B* were consistently identified regardless of stratification by neuropsychological dysfunction. Importantly, except for the within-gene interaction of *CACNG2*, all of the identified risk haplotypes and interactions involved SNPs from *DAO*.

**Conclusions:**

These results suggest that *DAO*, which is involved in the N-methyl-d-aspartate receptor regulation, signaling and glutamate metabolism, is the master gene of the genetic associations and interactions underlying schizophrenia. Besides, the interaction between *DAO* and *RASD2* has provided an insight in integrating the glutamate and dopamine hypotheses of schizophrenia.

## Introduction

Schizophrenia is a common, highly heritable, chronic mental disorder characterized by neuropsychological abnormalities and neurophysiological impairments [1], [2], [3]. The vulnerability to schizophrenia is influenced by a polygenic component, environmental factors, and their interactions [4]. Genes that confer vulnerability to schizophrenia have been identified in genetic studies [5], [6], [7], [8], but the genetic interactions among these genes and their interplay with neurobiological abnormalities and clinical subtypes of schizophrenia remains obscure.

There were relatively large number of genes located in various chromosome regions identified as schizophrenia candidate vulnerability genes from linkage studies [9], [10], [11], [12], positional cloning or candidate gene association studies [13], [14], [15], [16], and genome-wide association studies [17], [18], [19], [20]. Those schizophrenia vulnerability genes were collected in public databases such as SzGene [21] and SZGR [22]. However, these identified genes ran a replication and non-replication pattern. For example, association between schizophrenia and the Disrupted in Schizophrenia 1 (*DISC1*) gene was found and replicated in some genetic studies [23], [24], [25] but failed to replicate by some of the others [26], [27]. The inconsistency in gene findings came from various reasons such as population substructure/stratification, genetic heterogeneity, and problem of statistical power [28], [29]. In Taiwan, we have carried out a series of genetic studies using the single-ethnicity samples of Taiwan Chinese families of schizophrenia to search for the genomic regions in which potentially harbor vulnerability genes of schizophrenia using linkage analyses. We have further finely mapped the vulnerability genes of schizophrenia using positional cloning. Besides, we also carried out association studies to replicate the reported vulnerability genes in the literature from the other research groups.

The linkage studies for schizophrenia in Taiwan revealed suggestive linkage evidence of chromosome 1q42.1 [30], 6p24 [31], 8p21-12 [32], and 15q14 [33] using 52 families with at least two siblings affected with schizophrenia. We also found suggestive linkage evidence of 22q12 in an international collaborative study [34], where 557 families with at least two siblings affected with schizophrenia were recruited from the whole Taiwan. Genome-wide linkage analysis found the maximum nonparametric linkage z score was 2.88 for D10S2327 (100.92 cM) at 10q22.3 [35].

Using candidate-gene positional cloning approach we have finely mapped genes vulnerable to schizophrenia in Taiwan, including *DISC1* at chromosome 1q42 [36], *RASD2* and *CACNG2* at chromosome 22q12 [37], and *DPYSL2*, *TRIM35* and *PTK2B* at chromosome 8p21 (in preparation). Some genes are specifically associated with some subtypes of schizophrenia.

Besides, we have also performed the association studies in Taiwanese samples on *NRG1* (8p12), *DAO* (12q24), and *DAOA* (12q33.2) as these three genes were reported to have strong association with schizophrenia in the literature [38], [39], [40]. We did find significant association of *NRG1* [41] and *DAO* genes [42] with schizophrenia. However, we cannot find significant association of *DAOA* (*G72*) with schizophrenia [43]. In addition, the *LMBRD1* knockout mouse model revealed a neurological impairment and manifested a prominent social withdrawal behavior, representing the negative symptom state of schizophrenia. Moreover, we also found a significant association between *LMBRD1* gene and schizophrenia in our Taiwanese family samples of schizophrenia [44].

These 10 candidate genes found in our Taiwanese samples of schizophrenia did have active neurobiological functions in the central nervous system especially in neurotransmission and neurodevelopment. The *DAO* gene product is an enzyme that degrades the amino acid, d-Serine (d-Ser), which acts as a co-agonist at the glycine site of the N-methyl-d-aspartic acid (NMDA) receptor [45]. The *DAOA* gene product activates the DAO enzyme [46]. The biological functions of *DAO* and *DAOA* are involved in the hypothesized hypofunction of NMDA receptor complex as the potential pathogenesis of schizophrenia [47]. The *NRG1* gene product is a glycoprotein that interacts with the ERBB receptor tyrosine kinase. Moreover, *NRG1* mediates cell-to-cell interactions and plays a critical role in the development of the central nervous system [48]. The *DISC1* gene product is involved in neurite outgrowth and cortical development [49], [50]. The *LMBRD1* gene product is a lysosomal membrane protein involved in the transport and metabolism of cobalamin (vitamin B12), which plays a key role in maintaining neuronal function [51], [52]. The *DPYSL2* gene product, also known as collapsin response mediator protein-2 (CRMP-2), is a novel calmodulin-binding protein [53] that is differentially expressed in the anterior cingulate cortex of schizophrenic patients [54]. The *PTK2B* gene product, also known as proline-rich tyrosine kinase 2 (Pyk2), is involved in regulating calcium-induced ion channels and in cellular signaling by mitogen-activated protein kinase [55]. The *RASD2* gene product is a GTPase enriched in the striatum; the levels of this GTPase are reduced in dopamine super-sensitization [56]. The *CACNG2* gene product, also known as stargazin, is a subunit of the L-type calcium channel, and it modulates the calcium channel [57] and regulates the AMPA receptors [58]. The *TRIM35* gene product, also known as hemopoietic lineage switch 5 (*HLS5*), has been rarely studied with the exception of a report involving in cancer research [59].

Based on the specific protein functions of these candidate genes, we hypothesized that these vulnerability genes may involve in significant neurobiological functional pathways in neurotransmission and/or neurodevelopment, which may be responsible for the pathogenesis of schizophrenia. In order to examine the potential interactive effect of multiple genes found in our single-ethnicity schizophrenia samples, we carried out a direct sequencing study on these candidate genes to identify the potential functional single nucleotide polymorphisms (SNPs) in our Taiwan Chinese samples. These potential functional SNPs were then under this gene-gene interaction analyses.

The concept of intermediate phenotypes was introduced to refine the phenotypic characterization of schizophrenia and other psychiatric illnesses [60]. This strategy was also successfully applied in the exploration of the interactive effect of multiple genes using progressive brain change in schizophrenia [61]. We tested one potential intermediate phenotype: sustained attention as measured using a Continuous Performance Test (CPT) [62]. Deficits of sustained attention manifest not only in patients with schizophrenia but also in subjects with schizotypal personality disorder and in the nonpsychotic relatives of schizophrenic patients [63], [64], [65], [66], [67]. The normalized z score for d’, the sensitivity measure of sustained attention as assessed by the CPT, has been frequently used as a schizophrenia endophenotype. Schizophrenic patients with a z score below –2.5 are categorized as having a deficit in sustained attention. The recurrence risk ratio for schizophrenia among parents or siblings of this subgroup was higher than that for other schizophrenia probands [68], [69]. Thus, using performance on the CPT to define endophenotypes of impaired sustained attention among schizophrenics might help resolve the genetic heterogeneity observed in association studies of schizophrenia.

This study examined three hypotheses. First, that the within-gene and between-gene interactions are responsible for the complex genetics of schizophrenia. Second, that these interactions are moderated by degree of impairment of neuropsychological parameters, e.g., CPT deficits [62]. Third, that there exist comprehensible and testable neurobiological pathways which could be formulated based on the interactions of the functional expressions of these corresponding genes. Here we proposed a multi-stage procedure for identifying within-gene and between-gene interactions, and discussed the meaning of these interactions based on the glutamate and dopamine hypotheses of schizophrenia.

## Methods and Materials

### Ethics Statement

All potential patients who declined to participate or otherwise did not participate were eligible for treatment and were not disadvantaged in any other way by not participating in the study. In all cases, written informed consent was obtained after the study procedures were fully explained to subjects. The sample information and the data were de-identified before statistical data analysis. This study was approved by the Institutional Review Board of National Taiwan University Hospital.

### Study Subjects

Genomic DNA samples were collected from 912 schizophrenic patients (564 males and 348 females) and 600 normal controls (313 males and 287 females) in Taiwan’s Han Chinese population as part of four independent research programs. Of the 912 schizophrenic subjects, 702 were from multiplex families with at least two affected siblings, and these subjects were recruited as part of two research programs: the Multidimensional Psychopathology Study of Schizophrenia (MPSS) from 1993 to 2001 [70] and the Taiwan Schizophrenia Linkage Study (TSLS) from 1998 to 2002 [11], [35]. To ensure the independence of multiplex subjects, we randomly selected one affected sibling from each multiplex family for inclusion in the study. The other 210 schizophrenic subjects were from simplex families with only one affected sibling, and these subjects were recruited as part of another independent project. The 600 normal controls were selected from a representative supernormal genomic sample from Taiwan as part of a fourth independent project [71], with inclusion criteria of ≥60 years of age and a Short Portable Mental Status Questionnaire score of ≥14. Ninety-three MPSS families were interviewed by research psychiatrists using the Psychiatrist Diagnostic Assessment [72]. Six hundred and nine TSLS families were interviewed by well-trained assistants using the Mandarin Chinese version of the Diagnostic Interview for Genetic Studies (DIGS) [73]. The 210 patients from simplex families were interviewed using DIGS. For all subjects, the final diagnostic assessment was formulated by integrating either the Psychiatrist Diagnostic Assessment or the DIGS data with clinical information from medical records using the Specialist Diagnostic Assessment Sheet, based on the criteria of the Diagnostic and Statistical Manual of Mental Disorders, 4^th^ edition.

### Genomic Sequencing for Identifying New SNPs

A total of 50 normal controls, 50 multiplex patients and 50 simplex patients were randomly selected for sequencing. DNA sequences of promoters, exons, highly conserved introns, spliced variants, and isoforms of the ten schizophrenia candidate genes were amplified by polymerase chain reaction (PCR). PCR products were purified by treatment with exonuclease I and shrimp alkaline phosphatase (USB Corporation, Cleveland, OH, USA) and sequenced from both ends. DNA sequencing reactions were performed with BigDye Terminator Cycle Sequencing Version 3.1 (Applied Biosystems, Foster City, CA, USA) followed by analysis on an ABI 3730xl DNA Analyzer (Applied Biosystems). The resulting sequences were compared and aligned using the Polyphred Sequence Alignment Editor (http://droog.mbt.washington.edu/PolyPhred.htm/). Reference sequences were obtained from National Center for Biotechnology Information (NCBI) website (http://www.ncbi.nlm.nih.gov/).

SNPs satisfying one of the following conditions were selected for further genotyping: (1) SNPs in exons, (2) SNPs with a minor allele frequency (MAF) between 0 to 10% and an MAF difference between case and control groups of >0.02, (3) SNPs with MAF >10% and an MAF difference between case and control groups of >0.04, and (4) SNPs with a functional risk >3 of medium level, as defined in FastSNP [74]. We identified 99 SNPs for genotyping experiment: 17 SNPs in *DISC1* (1q42.1), 11 SNPs in *LMBRD1* (6q13), 14 SNPs in *DPYSL2* (8p22), 5 SNPs in *TRIM35* (8p22), 21 SNPs in *PTK2B* (8p22), 10 SNPs in *NRG1* (8p21), 5 SNPs in *DAO* (12q22), 6 SNPs in *G72* (13q32), 4 SNPs in *RASD2* (22q12), and 6 SNPs in *CACNG2* (22q12). The SNP annotation information, including rs number, region, function, and physical position, followed NCBI Build 36.3 (Table S1). Eighteen novel SNPs discovered from our direct DNA sequencing experiment were deposited in GenBank (http://www.ncbi.nlm.nih.gov/genbank/) (Table S2).

### SNP Genotyping

SNP genotyping was performed using the method of matrix-assisted laser desorption/ionization-time of flight mass spectrometry. PCR primers and genotyping probes that flanked the SNPs were designed using SpectroDESIGNER software (Sequenom, San Diego, CA, USA). A DNA fragment (100–300 bp) encompassing the SNP site was amplified using a PCR-ABI 9700 thermocycler (Applied Biosystems) according to the manufacturer’s instructions. After removing any deoxynucleotide triphosphate (dNTP) that was not incorporated and inactivating the shrimp alkaline phosphatase from the PCR product, primer extension was performed by adding the probe, Thermo Sequenase (Amersham Pharmacia, Piscataway, NJ, USA), and the appropriate dideoxynucleotide triphosphate (ddNTP)/dNTP mixture, followed by 55 cycles of denaturing at 94°C for 5 s, annealing at 52°C for 5 s, and extension at 72°C for 5 s. Sequenom’s SpectroPOINT matrix array was used to transfer the PCR products from the microplate to a 384-well SpectroCHIP. The mass spectrum from time-resolved spectra was retrieved using a MassARRAY mass spectrometer (Sequenom), and each spectrum was then analyzed using SpectroTYPER and SpectroREADER software (Sequenom) for genotype calling.

### Neuropsychological Assessment of Sustained Attention Using the CPT

Sustained attention was assessed using the unmasked and masked CPTs (Zd’ and Zmd’) [68]. The CPT indices of the normal controls were not measured. A CPT machine from Sunrise System, v. 2.20 (Pembroke, MA, USA) was used to conduct the tests for schizophrenic subjects [62]. Briefly, numerals from 0 to 9 were randomly presented on a screen for 50 ms each, at a rate of one per second. Subjects were asked to respond each time the numeral “9” was preceded by the numeral “1” on the screen. For the 25% masked session, a pattern of snow appeared on the screen to visually distort the images. Each test session began with 2 min of practice, which could be repeated if required. A total of 331 trials, with 34 (10%) target stimuli, were presented over a 5-min test session. A signal-detection index of performance on the test, sensitivity (d’), was derived from the hit rate (defined as the probability of a correct response to the target trials) and false-alarm rate (defined as the probability of a response to the non-target trials). Sensitivity measures an individual’s ability to discriminate target stimuli from non-target stimuli. In a 1-week test-retest reliability study, the intra-class correlation coefficients of reliability for d’ were 0.83 and 0.82 for the unmasked and the 25% masked tasks, respectively [68]. The effect of age, education, and sex on performance of the CPT was adjusted based on a community-based sample of 345 controls. The d’ was calculated as the Z-scores that were adjusted for these demographic characteristics [68]. For the unmasked CPT in this study, there were 455 (50%) patients with Zd’ ≥ –2.5, 298 (33%) patients with Zd’<–2.5, and 159 (17%) patients without Zd’ measurements. For the masked CPT, there were 357 (39%) patients with Zmd’ ≥ –2.5, 374 (41%) patients with Zmd’<–2.5, and 181 (20%) patients without Zmd’ measurements.

### Statistical Analyses

#### Quality control of samples and SNPs

The quality of the study samples and SNP markers was evaluated. Poor samples and SNPs were removed using a sequentially exclusive procedure. First, samples with a genotyping call rate (GCR) of <0.9 were removed. Next, SNPs with a GCR <0.9 in a case or control group were removed, and then SNPs with an MAF <0.01 were removed. Finally, SNPs with pFDR_HWE_ <0.05 were removed, where pFDR_HWE_ is an adjusted p-value that controls for the false-discovery rate (FDR) [75] in an exact Hardy-Weinberg equilibrium test [76] with 10,000 permutations in the control group. Finally, missing genotypes were imputed using BIMBAM software [77]. An unstratified analysis of overall samples and a stratified analysis of four CPT strata, Zd’<−2.5, Zd’ ≥ −2.5, Zmd’<−2.5, and Zmd’ ≥ −2.5, for genetic associations and interactions were conducted using the clean and imputed data.

#### Association tests

The permutation-based single-locus and multi-locus association tests were performed to examine genetic associations to schizophrenia. Here, 10,000 permutations were considered. For the single-locus association tests, genotype-based, allele-based, and trend-based association tests were performed using the CASECONTROL procedure in SAS/GENETICS software [78]. The HAPLOVIEW software [79] was used to measure coefficient of LD (*D*’) [80] of SNPs within candidate genes, and haplotype blocks were identified using Gabriel’s method [81]. Within each haplotype block, the overall haplotype likelihood-ratio association test and the individual haplotype likelihood-ratio association test [82] were conducted using the HAPLOTYPE procedure in SAS/GENETICS software [78]. An FDR [75] was calculated for corrections of multiple testing in each CPT stratum. A significant association was defined as a value of <0.05 for the adjusted p-values pFDR_SG_, pFDR_SA_, and pFDR_ST_, for genotype-based, allele-based, and trend-based single-locus association tests, respectively, and pFDR_H_ for the haplotype association test.

#### Gene-gene interaction analyses

A gene-based, multifactorial dimension reduction procedure was utilized to identify the within-gene and between-gene interactions with the highest testing accuracy based on a 10-fold cross validation procedure [83]. For identification of within-gene interactions, MDR software [84], [85], [86] was used to analyze all SNPs within a gene of study. The testing accuracy was calculated for all possible order-1 (i.e., a single SNP), order-2 (i.e., a pair of SNPs), order-3 (i.e., a triplet of SNPs), and order-4 (i.e., a quartet of SNPs) SNP combinations. The average testing accuracy of 10-fold cross validation samples was calculated. The model with the highest average testing accuracy was considered to be the best model. No statistical tests were performed at this stage. If the best model was an order-1 model, then we concluded that a within-gene interaction did not exist. Only the within-gene interactions with a cross-validation consistency ≥0.8 were regarded as candidates. Definitions of testing accuracy and cross-validation consistency can refer to the MDR User’s Guide.

A similar gene-based, multifactorial dimension reduction procedure was applied to each pair of genes to identify between-gene interactions. MDR software was used to analyze all SNPs within a pair of genes to determine the model with the highest average testing accuracy among all possible order-1, order-2, order-3, and order-4 SNP combinations. No statistical tests were performed at this stage. If the best model contained at least one SNP in the first gene and at least one SNP in the second gene, a between-gene interaction was found. Only the between-gene interactions with a cross-validation consistency ≥0.8 were regarded as candidates.

A permutation test was performed to validate the identified within-gene and between-gene interaction candidates using MDR-PT software [86]. Because we only needed to evaluate statistical significance of the interaction candidate (the best model of all possible order-1, order-2, order-3, and order-4 SNP combinations), in contrast to conventional interaction testing procedures, the number of tests was dramatically reduced in the gene-based MDR procedure. A 10-fold cross validation procedure was used, and 1,000 permutations were carried out to randomly exchange the case and control status of the samples. An empirical p-value of the testing accuracy for each of the identified interaction candidates was calculated. In unstratified analysis and CPT-stratified analysis, only the within-gene and between-gene interaction candidates having an FDR-adjusted empirical p-value (i.e., pFDR_WG_ for within-gene interaction and pFDR_BG_ for between-gene interaction) of <0.05 were regarded as confirmed and reliable interactions. In this paper, notation pFDR denotes an FDR-adjusted p-value for corrections of multiple testing.

## Results

After the direct sequencing analysis, ninety-nine SNPs were qualified for the genotyping study in these ten candidate genes. The quality control procedures first removed 65 samples with a GCR <0.9. Next, 11 SNPs with a GCR <0.9 in the case or control group were removed, and 4 SNPs with an MAF <0.01 were removed; no SNPs had a pFDR_HWE_ <0.05. After the quality control procedures, 84 SNPs remained including 15 SNPs in *DISC1*, 7 SNPs in *LMBRD1*, 10 SNPs in *DPYSL2*, 4 SNPs in *TRIM35*, 21 SNPs in *PTK2B*, 9 SNPs in *NRG1*, 5 SNPs in *DAO*, 4 SNPs in *G72*, 3 SNPs in *RASD2*, and 6 SNPs in *CACNG2*, from 1,447 samples from 893 cases and 554 controls. Finally, missing genotypes (∼0.57% genotypes of all data) were imputed.

**Figure 1:**
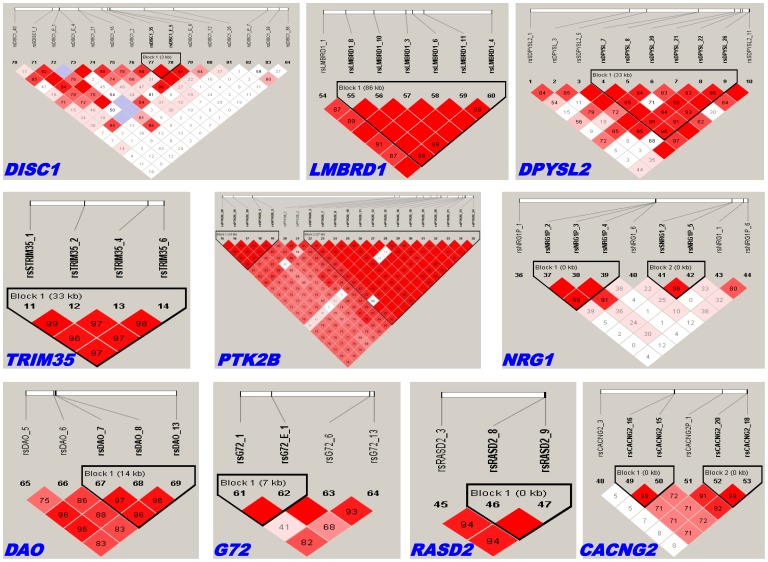
The LD structures of 84 SNPs in 10 candidate genes for vulnerability to schizophrenia. For each gene, the id of each SNP in the gene is listed, and the locations reflect the relative physical positions of the SNPs (in units of base pairs). The LD coefficient, D’, is provided unless D’ = 1. The color scheme for D’ presentation is as follows: white depicts the case of D’ <1 and LOD <2; blue depicts the case of D’ = 1 and LOD <2; pink or light red depicts the case of D’ <1 and LOD ≥2; bright red depicts the case of D’ = 1 and LOD ≥2. The LD block(s) within each gene is marked by an inverted triangle based on Gabriel’s method [81].

**Figure 2:**
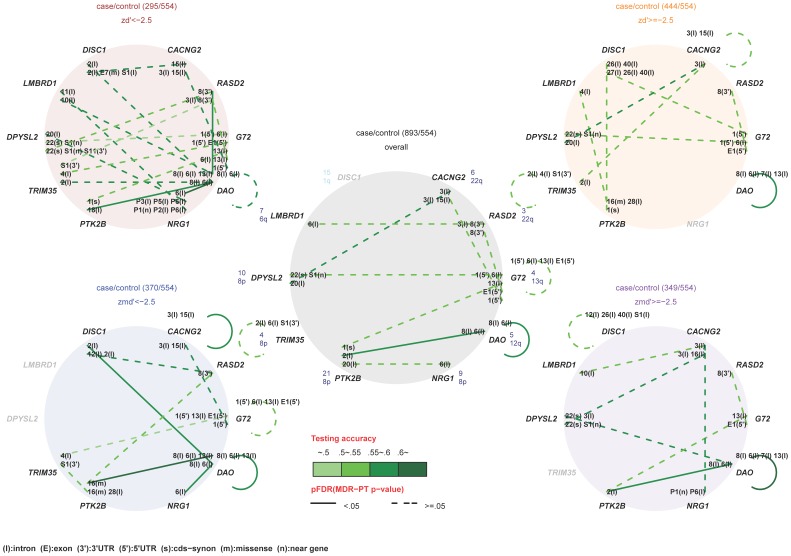
Within-gene and between-gene interactions in schizophrenia for the unstratified and CPT-stratified interaction analyses. The unstratified interaction plot is at the center, and the CPT-stratified plots with the stratum name are located in the four corners. Stratum name and sample size of cases and controls are provided in the title for each panel. Gene name, the number of SNPs in the gene, and the located chromosome are provided around each circle. If an interaction was identified, the SNP id along with its location in the gene (in parentheses) are provided. Abbreviations for SNP locations are: 3′, 3′ untranslated region; 5′, 5′ untranslated region; I, intron; E, exon; s, synonymous SNP; n, near-gene SNP; m, missense SNP. Interaction candidates with a pFDR <0.05 and ≥0.05 are connected by a solid line and dashed line, respectively, and the level of testing accuracy is represented by the line color from light green (less accurate) to dark green (highly accurate).

First, unstratified analyses were performed. Genotype-, allele-, and trend-based single-locus association tests consistently identified rsCACNG2_3 and rsDAO_13 as significantly associated with schizophrenia, but the significance did not survive a correction for multiple testing (Table S1). Haplotype association tests were conducted for the following genes (the number of LD blocks is indicated in parentheses): *DISC1* (1), *LMBRD1* (1), *DPYSL2* (1), *TRIM35* (1), *PTK2B* (2), *NRG1* (2), *DAO* (1), *G72* (1), *RASD2* (1), and *CACNG2* (2) (Figure 1). Only the risk haplotype *A*-*T*-*C* of the SNP triplet rsDAO7-rsDAO8-rsDAO13 within the LD block of *DAO* was significantly associated with schizophrenia (pFDR_H_ = 0.0090; Table S3). No controls carried this risk haplotype, but 1.24% of schizophrenia subjects carried this haplotype. We found a within-gene interaction of DAO, rsDAO_6*rsDAO_8 (pFDRWG = 0.0028; testing accuracy = 0.5811), and a between-gene interaction of DAO and PTK2B, rsDAO_6*rsDAO_8*rsPTK2B_2 (pFDRBG = 0.0028, testing accuracy = 0.5940; Figure 2). The cross-validation accuracy of the two identified interactions was 100%.

Second, CPT-stratified analyses were performed. Single-locus association tests only found a borderline significance of rsCACNG2_3 in the subgroup “zmd’<−2.5” (pFDRST = 0.042) after a multiple test correction (Table S1). The frequencies of the risk allele *T* were 0.3595 and 0.2825, and their standard errors were 0.0179 and 0.0136, in the case and control groups, respectively. The LD structures of the four CPT strata differed only slightly for *DPSYL2* and *PTK2B* when compared with the results of the non-stratified analysis (Figures S1, S2, S3, S4 and Figure 1). All haplotype association tests in the four CPT strata identified a risk haplotype *A*-*T*-*C* of the SNP triplet rsDAO7-rsDAO8-rsDAO13 within the LD block of *DAO* (pFDR_H_ = 0.0034 for zd’ ≥ −2.5; pFDRH = 0.0090 for zd’<−2.5; pFDRH = 0.0047 for zmd’ ≥ −2.5; pFDRH = 0.0044 for zmd’<−2.5; Table S3). Approximately 1−2% of schizophrenia subjects carried this haplotype, whereas none of the control subjects did.

In the zd’<−2.5 stratum we found several between-gene interactions: rsDAO_6*rsDAO_8*rsPTK2B_18 (pFDRBG = 0.0312; testing accuracy = 0.5800), rsDAO6*rsDAO8*rsNRG1_6 (pFDRBG = 0.0043; testing accuracy = 0.6222), and rsDAO_6*rsDAO_8*rsDAO_13*rsRASD2_8 (pFDRBG = 0.0043; testing accuracy = 0.5911). The analysis of the zd’ ≥ −2.5 stratum identified the within-gene interaction rsDAO_6*rsDAO_7*rsDAO_8*rsDAO_13 (pFDRWG = 0.0150; testing accuracy = 0.5842). The analysis of the zmd’<−2.5 stratum identified the within-gene interactions rsDAO_6*rsDAO_8*rsDAO_13 (pFDRWG = 0.0018; testing accuracy = 0.5882) and rsCACNG2_3*rsCACNG2_15 (pFDRWG = 0.0297; testing accuracy = 0.5597), and the between-gene interactions rsDAO_6*rsDAO_8*rsDISC1_2 (pFDRBG = 0.0069; testing accuracy = 0.5917), rsDAO_6*rsDAO_8*rsDAO_13*rsPTK2B_16 (pFDRBG = 0.0018; testing accuracy = 0.6020), and rsDAO_6*rsDAO_8*rsNRG1_6 (pFDRBG = 0.0018; testing accuracy = 0.5935). The analysis of the zmd’ ≥ −2.5 stratum identified the within-gene interaction rsDAO_6*rsDAO_7*rsDAO_8*rsDAO_13 (pFDRWG = 0.0045; testing accuracy = 0.6015) and the between-gene interaction rsDAO_6*rsDAO_8*rsPTK2B_2 (pFDRBG = 0.0158; testing accuracy = 0.5842; Figure 2). All of the identified interactions had a cross-validation accuracy of ≥0.8.

## Discussion

Results from the single-locus association tests suggest that single SNPs have little impact on schizophrenia. Only borderline significance was found for a single SNP, rsCACNG2_3. Still, a future study with a larger sample size might indeed identify minor-effect SNPs associated with schizophrenia. By incorporating LD information, our haplotype analysis identified a risk haplotype *A*-*T*-*C* of the SNP triplet rsDAO7-rsDAO8-rsDAO13 on *DAO*. The low frequency of this risk haplotype suggests that a common disease can be caused by rare variants with a large impact (common disease, rare variant) [87]. The finding is worthy of a further investigation by a recruitment of more samples and by an independent replication using another cohort in the future.

Multiple within-gene and between-gene interactions were identified. Genetic interaction analysis found that *DAO* is a master gene in the genetic interaction network underlying schizophrenia. Among the confirmed and reliable genetic interactions, all the within-gene interactions were found in *DAO*, except for a within-gene interaction in *CACNG2*, and all the between-gene interaction pairs involved *DAO*. Two master SNP nodes, rsDAO_6 and rsDAO_8, play key roles in the within- and between-gene interactions. Moreover, *DAO* and *PTK2B* were identified in most of the unstratified and stratified interaction analyses as an important between-gene interaction pair. These results support our hypothesis that within-gene and between-gene interactions may be responsible for the complex genetics of schizophrenia.

Our analysis shows that *DAO* plays an important role in the genetic associations and interactions for schizophrenia. The *DAO* gene product is involved in the signal transduction pathway of the NMDA receptor [88], [89]. Substrates of the *DAO*-encoded enzyme, especially d-Ser, may bind to the glycine site of the NMDA receptor and function as a co-agonist [45], [90]; thus, the *DAO* gene product may regulate the NMDA receptor by the level of d-Ser, thereby opening the calcium channel of the NMDA receptor. d-Ser was found to inhibit the AMPA receptor–mediated current in rat hippocampal neurons [91]; thus, the *DAO* gene product was implicated in the pathogenesis of schizophrenia [89].

The CPT-stratified analyses indicated that the genetic interactions of risk SNPs for schizophrenia were moderated by the degree of sustained attention deficit among schizophrenics. The within-gene interaction of *DAO* and the between-gene interaction of *DAO* and *PTK2B* were found for almost all unstratified and CPT-stratified analyses; however, some stratum-specific genetic interactions were found in the severe sustained attention deficit groups (zd’<−2.5 and zmd’<−2.5). We identified the between-gene interaction pair DAO*PTK2B, DAO*NRG1 and DAO*RASD2 in stratum zd’<−2.5, and the between-gene interaction pair DAO*NRG1, DAO*DISC1, and DAO*PTK2B in stratum zmd’<−2.5. These results support our hypothesis that the degree of sustained attention deficit moderates interactions of risk SNPs for schizophrenia.

The glutamate receptor, such as NMDA receptor, can be triggered by d-Ser, which is a co-agonist of NMDA receptor. Activating this receptor will induce the calcium influx, which may cause clustering and autophosphorylation of Pyk2 (Proline-rich tyrosine kinase 2, which is also known as Ptk2b) in the postsynaptic neuron [92]. This cluster can be disrupted by the action of striatal-enriched protein-tyrosine phosphatase (STEP), which in turn down-regulate the long term potentiation (LTP) [93]. Affecting the long-term potentiation early in development in the hippocampus CA1 region may contribute to cognitive deficits observed later in schizophrenia [94]. Therefore, decreasing the concentration of d-Ser may lead to Schizophrenia.

**Figure 3:**
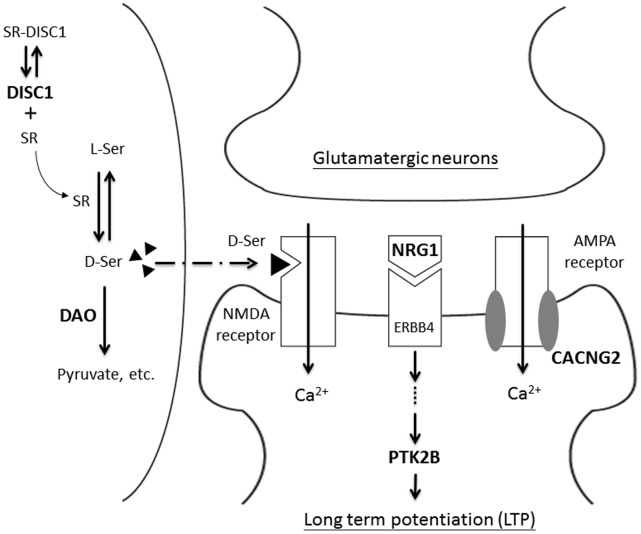
A schematic diagram to show the relation among genes that involved in the observed gene-gene interactions. The top, bottom, and left side components represent the pre-synaptic neuron, post-synaptic neuron, and glial cells, respectively. Three types of receptors (NMDA receptor, ERBB4 receptor, and AMPA receptor) are drawn on the post-synaptic neuron. All the genes involved in the gene-gene interactions are shown in boldface. *NRG1* is the ligand for ERBB4 receptor, which may trigger the long term potentiation by way of the *PTK2B* protein activation. *DISC1* may stabilize serine racemase (SR), which will convert L-Serine to d-Serine (d-Ser). The black triangle on the side of NMDA receptor represents d-Ser, which is a co-agonist of this receptor. Both NMDA and AMPA receptors are calcium channels, which may increase calcium influx upon activation. Two molecules of *CACNG2* (shown by the grey oval) were found on each AMPA receptor. Even though *CACNG2* is a subunit of AMPA receptor, this protein is explicitly drawn in order to show its role in gene-gene interaction.

As shown in Figure 3, the concentration of d-Ser is regulated by two factors. First of all, serine racemase (SR) catalyzes the conversion of L-Ser to d-Ser, which increases the d-Ser concentration in the synapse area. On the other hand, *DAO* gene encodes an enzyme that degrades d-Ser. The inactivation of *SR* or the activation of *DAO* may decrease the d-Ser concentration and thus decrease calcium influx. This argument is consistent with the observations that polymorphisms of *SR* and *DAO* genes were implicated as risk factors for schizophrenia [95], [96], respectively).

It has been shown that a mutant Disc1 failed to bind and stabilize *SR*. As a result, the concentration of d-Ser is lowered in mouse model [97]. The effect of this d-Ser deficit can be further enhanced by having a polymorphism, which may have a higher *DAO* activity. In other words, the cognitive deficits are expected to be even more severe when the *DISC1* and *DAO* polymorphisms co-existed in the same cell.

Because the active D-amino acid oxidase is a dimer [98], the hetero-dimer of a mutant and a wild type protein may have the mutant phenotype. On the basis of the generalized model of gene dosage and dominant negative effects in macromolecular complexes [99], about 25% of the dimers are wild type dimers. If, however, there are two polymorphisms within the *DAO* gene co-exist in trans, none of the dimers are wild type *DAO* proteins. It appeared that one polymorphism enhances the effect of the other polymorphism. In other words, these two polymorphisms are interacting with each other within the *DAO* gene. The association of the polymorphism of *CACNG2* and schizophrenia [37] may be related to the calcium influx controlled by the AMPA receptor. *CACNG2* protein regulates synaptic targeting of AMPA receptors (AMPAR) [100], because *CACNG2* (also known as stargazin) is a subunit of AMPAR. Since there are two *CACNG2* proteins on an active AMPAR (see Figure 3), the within gene interaction of *CACNG2* can also be explained by using the generalized model [99].

As described in the introduction, the polymorphisms of *NRG1* and *PTK2B* are associated with schizophrenia, respectively. It is interesting to note that neuregulin 1 (Nrg1) activates Fyn and Pyk2 (Ptk2b) kinases and then modulates channel properties of NMDA receptor [101] in mouse model. If NMDA receptor is regulated by both *NRG1* and *DAO* proteins, the *NRG1* may enhance the down-regulation effect of *DAO* protein on calcium influx. Thus, a variation that attenuates the activity of either *NRG1* or *PTK2B* (human orthologs of Nrg1 and Pyk2) is likely to enhance the cognitive deficits caused by *DAO* protein. This synergistic effect is similar to the synthetic lethal phenomenon [102]. Because *PTK2B* protein is in the downstream of *NRG1* signaling pathway (see Figure 3), the effect *PTK2B* on cognitive deficits might be more direct than that of *NRG1*. This may explain why the interaction of *DAO* and *PTK2B* was observed regardless of stratification by neuropsychological dysfunction.

In addition to the glutamate hypothesis, the dopamine hypothesis of has also been used to explain the possible cause of schizophrenia. In fact, the striatal dopamine abnormalities are now clearly demonstrated in patients with schizophrenia and at risk population (reviewed in [103]). Interestingly, the protein product of *RASD2* (also known as Rhes, Ras homolog enriched in striatum) is a risk factor for schizophrenia [37]. This protein has been implicated in modulating dopamine signaling in striatal medium spiny neurons [104]. Furthermore, dopamine receptors D2 might associate with schizophrenia via Akt signaling [105]. It has been shown that *RASD2* may regulate the Akt pathway by interacting with the regulatory subunit of *PI3K* [106]. This observation provides a possible molecular mechanism for the association of *RASD2* polymorphism and schizophrenia. The striatum is an area where glutamatergic input from cerebral cortex and a dopaminergic input from the substantia nigra come together. Thus, the interaction between *DAO* and *RASD2* genes suggests a possible link between dopamine hypothesis [107] and glutamate hypothesis [108].

Social-demographic-matched case-control design was favorable but not considered before. The main reason was that the available schizophrenic patients and normal controls in this study were not affordable for a complete sample matching by all important social-demographic variables such as gender, age, and education level. In order to avoid false-positive findings, we therefore examined if gender, age and education level were confounders. The post-analyses of the unstratified data of overall samples and the stratified data of CPT strata showed that all the SNPs didn’t have significantly different genotypic distributions in gender, age and education level groups. The least FDR-adjusted p-value for the association between SNPs and gender, age and education level groups was 0.252, 0.202 and 0.145, respectively. The results suggest that gender, age and education level were not confounders to the outcomes of genetic association and interaction in this study.

We also performed logistic regression analysis to examine the interactions reported by our gene-based MDR procedure. Logistic regression, a parametric model, has advantages in exploring genotype-phenotype relationship but suffering from the curse of dimensionality [84]. Most of our analyses either met a convergence failure while solving maximum likelihood estimates of regression coefficients or the fitted models failed to pass a lack-of-fit test [109] especially for the models involving more variables and higher order interactions. Among the genetic interactions reported by the gene-based MDR, logistic regression can fit well in two cases. The first case was about the within-gene interaction of the *DAO* gene in an unstratified analysis. The raw p-values from a type III analysis of rsDAO_8, rsDAO_6 and their interaction rsDAO_8*rsDAO_6 were 0.9994, 0.9995, and 0.0000 for nominal genotype coding and 0.0002, 0.0289, and 0.0000 for continuous genotype coding. In this case, both the logistic regression and gene-based MDR analysis identified the within-gene interaction of the *DAO* gene.

The second case was about the within-gene interaction of the *CACNG2* gene in the zmd’<-2.5 stratum. The raw p-values from a type III analysis for rsCACNG2_3, rsCACNG2_15 and their interaction rsCACNG2_3*rsCACNG2_15 were 0.0023, 0.8214, and 0.3651 for nominal genotype coding and 0.0178, 0.5757, and 0.8600 for continuous genotype coding. SNP rsCACNG2_3 was the only SNP showing a marginal significance of genetic association in this study. However, logistic regression analysis did not identify the interaction rsCACNG2_3*rsCACNG2_15 reported by the gene-based MDR. The discrepancy between the results of the gene-based MDR and logistic regression analyses may cause by a complex pattern of genetic interaction of rsCACNG2_3 and rsCACNG2_15. Compared with the low-risk genotype rsCACNG2_3 = *GG*, genotypes rsCACNG2_3 = *TG* and *TT* are high-risk except that the genotype combinations rsCACNG2_3 = *TG* and rsCACNG2_15 = *GA* are low-risk. Therefore, in our gene-based MDR analysis, the interaction model of rsCACNG2_3 and rsCACNG2_15 showed a higher testing accuracy than the main effect model of rsCACNG2_3. Whatever a 4-degree-of-freedom type III interaction test or four 1-degree-of-freedom interaction tests using dummy variables, logistic regression analysis requires more samples in order to detect such a complex pattern of genetic interaction.

This study is limited, however, by the lack of CPT data for the normal controls. The results of the CPT analysis rely on the assumption that the distribution of CPT scores is relatively homogeneous in normal controls–an assumption that is held generally for the Taiwan Han Chinese population [68]. Besides, this study is limited by the adoption of only ten candidate vulnerability genes found in the Taiwanese population, and not including all other candidate genes reported in literature. However, we did also take the strength of single-ethnicity samples in this study to reduce false-positive and/or false-negative results due to genetic heterogeneity. Finally, this study is also limited by no replications for the reported genetic association and interaction. The findings reported in this study will be replicated in our ongoing genome-wide association study of schizophrenia in the Taiwan Han Chinese population.

This study identified statistically significant within-gene and between-gene interactions in schizophrenia, and it thereby provides a new paradigm for exploring genetic pathways underlying this disease. Furthermore, this study also revealed multiple neurobiological pathways responsible for the multiple pathogenesis mechanisms of schizophrenia potentially. Further studies are necessary to explore the molecular mechanisms underlying these gene-gene interactions by bridging clinical phenotypes of schizophrenia, neurobiological abnormalities, genetic expressions, and the associated/interactive genes. Identification of interactions involving SNPs on more than two genes requires a large sample size and will be conducted after our sample size has been further increased.

## Supporting Information

Figure S1 **The LD structures of 84 SNPs in 10 candidate genes for the Zd’ ≥ −2.5 stratum.** For each gene, the id of each SNP in the gene is listed, and the locations reflect the relative physical positions of the SNPs (in units of base pairs). The LD coefficient, D’, is provided unless D’ = 1. The color scheme for D’ presentation is as follows: white depicts the case of D’ <1 and LOD <2; blue depicts the case of D’ = 1 and LOD <2; pink or light red depicts the case of D’ <1 and LOD ≥2; bright red depicts the case of D’ = 1 and LOD ≥2. The LD block(s) within each gene is marked by an inverted triangle based on Gabriel’s method [81].
(TIF)

Figure S2 **The LD structures of 84 SNPs in 10 candidate genes for the Zd’<−2.5** **stratum.** For each gene, the id of each SNP in the gene is listed, and the locations reflect the relative physical positions of the SNPs (in units of base pairs). The LD coefficient, D’, is provided unless D’ = 1. The color scheme for D’ presentation is as follows: white depicts the case of D’ <1 and LOD <2; blue depicts the case of D’ = 1 and LOD <2; pink or light red depicts the case of D’ <1 and LOD ≥2; bright red depicts the case of D’ = 1 and LOD ≥2. The LD block(s) within each gene is marked by an inverted triangle based on Gabriel’s method [81].
(TIF)

Figure S3 **The LD structures of 84 SNPs in 10 candidate genes for the Zmd’ ≥ −2.5** **stratum.** For each gene, the id of each SNP in the gene is listed, and the locations reflect the relative physical positions of the SNPs (in units of base pairs). The LD coefficient, D’, is provided unless D’ = 1. The color scheme for D’ presentation is as follows: white depicts the case of D’ <1 and LOD <2; blue depicts the case of D’ = 1 and LOD <2; pink or light red depicts the case of D’ <1 and LOD ≥2; bright red depicts the case of D’ = 1 and LOD ≥2. The LD block(s) within each gene is marked by an inverted triangle based on Gabriel’s method [81].
(TIF)

Figure S4 **The LD structures of 84 SNPs in 10 candidate genes for the Zmd’<−2.5** **stratum.** For each gene, the id of each SNP in the gene is listed, and the locations reflect the relative physical positions of the SNPs (in units of base pairs). The LD coefficient, D’, is provided unless D’ = 1. The color scheme for D’ presentation is as follows: white depicts the case of D’ <1 and LOD <2; blue depicts the case of D’ = 1 and LOD <2; pink or light red depicts the case of D’ <1 and LOD ≥2; bright red depicts the case of D’ = 1 and LOD ≥2. The LD block(s) within each gene is marked by an inverted triangle based on Gabriel’s method [81].
(TIF)

Table S1 **Results of single-locus association tests.** Chromosome, gene name, SNP ID, rs number, region, function, and physical position are shown. The raw p-values and FDR-adjusted p-values are shown for genotype-based (X_G), allele-based (X_A), and trend-based (X_T) association tests in an unstratified analysis of overall samples and a stratified analysis of four CPT strata, Zd’ ≥ −2.5, Zd’<−2.5, Zmd’ ≥ −2.5, and Zmd’<−2.5.
(PDF)

Table S2 **Novel SNPs discovered from DNA sequencing.** Observation, chromosome, gene name, SNP ID, rs number, and ss number are shown. Eighteen novel SNPs discovered from our direct DNA sequencing experiment were deposited in GenBank. Their ss numbers are listed in the last column of this table.
(XLSX)

Table S3 **Results of haplotype-based association tests.** The results of haplotype-based association tests in an unstratified analysis of overall samples and a stratified analysis of four CPT strata, Zd’ ≥ −2.5, Zd’<−2.5, Zmd’ ≥ −2.5, and Zmd’<−2.5, are shown separately. In each sub-table, gene, LD block, haplotype, haplotype frequencies in case, control and combined groups, and the exact p-values and FDR-adjusted p-values of haplotype-based association tests are provided.
(PDF)

## References

[1] Bilder RM, Goldman RS, Robinson D, Reiter G, Bell L, et al (2000) Neuropsychology of first-episode schizophrenia: Initial characterization and clinical correlates. American Journal of Psychiatry 157: 549–559. 10739413 10.1176/appi.ajp.157.4.549

[2] Cosway R, Byrne M, Clafferty R, Hodges A, Grant E, et al (2000) Neuropsychological change in young people at high risk for schizophrenia: results from the first two neuropsychological assessments of the Edinburgh High Risk Study. Psychological Medicine 30: 1111–1121. 12027047 10.1017/s0033291799002585

[3] Thaker GK, Ross DE, Buchanan RW, Adami HM, Medoff DR (1999) Smooth pursuit eye movements to extra-retinal motion signals: deficits in patients with schizophrenia. Psychiatry Research 88: 209–219. 10622341 10.1016/s0165-1781(99)00084-0

[4] Risch N, Baron M (1984) Segregation analysis of schizophrenia and related disorders. American Journal of Human Genetics 36: 1039–1059. 6496472 PMC1684519

[5] International Schizophrenia Consortium, Purcell SM, Wray NR, Stone JL, Visscher PM, et al (2009) Common polygenic variation contributes to risk of schizophrenia and bipolar disorder. Nature 460: 748–752. 19571811 10.1038/nature08185 PMC3912837

[6] Stefansson H, Ophoff RA, Steinberg S, Andreassen OA, Cichon S, et al (2009) Common variants conferring risk of schizophrenia. Nature 460: 744–747. 19571808 10.1038/nature08186 PMC3077530

[7] O’Donovan MC, Williams NM, Owen MJ (2003) Recent advances in the genetics of schizophrenia. Human Molecular Genetics 12 Spec No 2: R125–R133. 10.1093/hmg/ddg302 12952866

[8] Owen MJ, Williams NM, O’Donovan MC (2004) The molecular genetics of schizophrenia: Findings promise new insights. Molecular Psychiatry 9: 14–27. 14581932 10.1038/sj.mp.4001444

[9] Levinson DF, Holmans P, Straub RE, Owen MJ, Wildenauer DB, et al (2000) Multicenter linkage study of schizophrenia candidate regions on chromosomes 5q, 6q, 10p, and 13q: schizophrenia linkage collaborative group III. American Journal of Human Genetics 67: 652–663. 10924404 10.1086/303041 PMC1287525

[10] Mowry BJ, Holmans PA, Pulver AE, Gejman PV, Riley B, et al (2004) Multicenter linkage study of schizophrenia loci on chromosome 22q. Molecular Psychiatry 9: 784–795. 15007391 10.1038/sj.mp.4001481

[11] Hwu HG, Faraone SV, Liu CM, Chen WJ, Liu SK, et al (2005) Taiwan schizophrenia linkage study: the field study. American Journal of Medical Genetics Part B (Neuropsychiatric Genetics) 134: 30–36. 10.1002/ajmg.b.30139 15685625

[12] Ng MY, Levinson DF, Faraone SV, Suarez BK, DeLisi LE, et al (2009) Meta-analysis of 32 genome-wide linkage studies of schizophrenia. Molecular Psychiatry 14: 774–785. 19349958 10.1038/mp.2008.135 PMC2715392

[13] Gurling HM, Critchley H, Datta SR, McQuillin A, Blaveri E, et al (2006) Genetic association and brain morphology studies and the chromosome 8p22 pericentriolar material 1 (PCM1) gene in susceptibility to schizophrenia. Archives of General Psychiatry 63: 844–854. 16894060 10.1001/archpsyc.63.8.844 PMC2634866

[14] Carroll LS, Williams NM, Moskvina V, Russell E, Norton N, et al (2010) Evidence for rare and common genetic risk variants for schizophrenia at protein kinase C, alpha. Molecular Psychiatry 15: 1101–1011. 19786960 10.1038/mp.2009.96

[15] Shi J, Gershon ES, Liu C (2008) Genetic associations with schizophrenia: meta-analyses of 12 candidate genes. Schizophrenia Research 104: 96–107. 18715757 10.1016/j.schres.2008.06.016 PMC2562556

[16] Greenwood TA, Light GA, Swerdlow NR, Radant AD, Braff DL (2012) Association analysis of 94 candidate genes and schizophrenia-related endophenotypes. PLoS ONE 7: e29630. 22253750 10.1371/journal.pone.0029630 PMC3258248

[17] Potkin SG, Turner JA, Guffanti G, Lakatos A, Fallon JH, et al (2009) A genome-wide association study of schizophrenia using brain activation as a quantitative phenotype. Schizophrenia Bulletin 35: 96–108. 19023125 10.1093/schbul/sbn155 PMC2643953

[18] Ripke S, Sanders AR, Kendler KS, Levinson DF, Sklar P, et al (2011) Genome-wide association study identifies five new schizophrenia loci. Nature Genetics 43: 969–976. 21926974 10.1038/ng.940 PMC3303194

[19] Yue WH, Wang HF, Sun LD, Tang FL, Liu ZH, et al (2011) Genome-wide association study identifies a susceptibility locus for schizophrenia in Han Chinese at 11p11.2. Nature Genetics 43: 1228–1231. 22037552 10.1038/ng.979

[20] Yamada K, Iwayama Y, Hattori E, Iwamoto K, Toyota T, et al (2011) Genome-wide association study of schizophrenia in Japanese population. PLoS ONE 6: e20468. 21674006 10.1371/journal.pone.0020468 PMC3108953

[21] Allen NC, Bagade S, McQueen MB, Ioannidis JPA, Kavvoura FK, et al (2008) Systematic meta-analyses and field synopsis of genetic association studies in schizophrenia: the SzGene database. Nature Genetics 40: 827–834. 18583979 10.1038/ng.171

[22] Jia P, Sun J, Guo AY, Zhao Z (2010) SZGR: a comprehensive schizophrenia gene resource. Molecular Psychiatry 15: 453–462. 20424623 10.1038/mp.2009.93 PMC2861797

[23] Hodgkinson CA, Goldman D, Jaeger J, Persaud S, Kane JM, et al (2004) Disrupted in schizophrenia 1 (DISC1): association with schizophrenia, schizoaffective disorder, and bipolar disorder. American Journal of Human Genetics 75: 862–872. 15386212 10.1086/425586 PMC1182115

[24] Thomson PA, Wray NR, Millar JK, Evans KL, Hellard SL, et al.. (2005) Association between the TRAX/DISC locus and both bipolar disorder and schizophrenia in the Scottish population. Molecular Psychiatry 10: 657–668, 616. 10.1038/sj.mp.4001669 15838535

[25] Zhang X, Tochigi M, Ohashi J, Maeda K, Kato T, et al (2005) Association study of the DISC1/TRAX locus with schizophrenia in a Japanese population. Schizophrenia Research 79: 175–180. 16039834 10.1016/j.schres.2005.05.023

[26] Chen QY, Chen Q, Feng GY, Lindpaintner K, Wang LJ, et al (2007) Case-control association study of Disrupted-in-Schizophrenia-1 (DISC1) gene and schizophrenia in the Chinese population. Journal of Psychiatric Research 41: 428–434. 16524593 10.1016/j.jpsychires.2006.01.001

[27] Mathieson I, Munafo MR, Flint J (2011) Meta-analysis indicates that common variants at the DISC1 locus are not associated with schizophrenia. Molecular Psychiatry. 10.1038/mp.2011.41 PMC3359642 21483435

[28] Cardon LR, Bell JI (2001) Association study designs for complex diseases. Nature Reviews Genetics 2: 91–99. 10.1038/35052543 11253062

[29] Wang WYS, Barratt BJ, Clayton DG, Todd JA (2005) Genome-wide association studies: Theoretical and practical concerns. Nature Reviews Genetics 6: 109–118. 10.1038/nrg1522 15716907

[30] Hwu HG, Liu CM, Fann CSJ, Ou-Yang WC, Lee SC (2003) Linkage of schizophrenia with chromosome 1q loci in Taiwanese families. Molecular Psychiatry 8: 445–452. 12740602 10.1038/sj.mp.4001235

[31] Hwu HG, Lin MW, Lee PC, Lee SFC, Ou-Yang WC, et al (2000) Evaluation of linkage of markers on chromosome 6p with schizophrenia in Taiwanese families. American Journal of Medical Genetics 96: 74–78. 10686556

[32] Liu C-M, Hwu H-G, Fann CSJ, Lin C-Y, Liu Y-L, et al (2005) Linkage evidence of schizophrenia to loci near neuregulin 1 gene on chromosome 8p21 in Taiwanese families. American Journal of Medical Genetics Part B: Neuropsychiatric Genetics 134B: 79–83. 10.1002/ajmg.b.20161 15704228

[33] Liu CM, Hwu HG, Lin MW, Ou-Yang WC, Lee SFC, et al (2001) Suggestive evidence for linkage of schizophrenia to markers at chromosome 15q13–14 in Taiwanese families. American Journal of Medical Genetics 105: 658–661. 11803511 10.1002/ajmg.1547

[34] Gill M, Vallada H, Collier D, Sham P, Holmans P, et al (1996) A combined analysis of D22S278 marker alleles in affected sib-pairs: Support for a susceptibility locus for schizophrenia at chromosome 22q12. American Journal of Medical Genetics 67: 40–45. 8678112 10.1002/(SICI)1096-8628(19960216)67:1<40::AID-AJMG6>3.0.CO;2-W

[35] Faraone SV, Hwu HG, Liu CM, Chen WJ, Tsuang MM, et al (2006) Genome scan of Han Chinese schizophrenia families from Taiwan: confirmation of linkage to 10q22.3. American Journal of Psychiatry 163: 1760–1766. 17012687 10.1176/ajp.2006.163.10.1760

[36] Liu Y-L, Fann CS-J, Liu C-M, Chen WJ, Wu J-Y, et al (2006) A single nucleotide polymorphism fine mapping study of chromosome 1q42.1 reveals the vulnerability genes for schizophrenia, GNPAT and DISC1: Association with impairment of sustained attention. Biological Psychiatry 60: 554–562. 16997000 10.1016/j.biopsych.2006.04.024

[37] Liu YL, Fann CS, Liu CM, Chen WJ, Wu JY, et al (2008) RASD2, MYH9, and CACNG2 genes at chromosome 22q12 associated with the subgroup of schizophrenia with non-deficit in sustained attention and executive function. Biological Psychiatry 64: 789–796. 18571626 10.1016/j.biopsych.2008.04.035

[38] Nicodemus KK, Law AJ, Luna A, Vakkalanka R, Straub RE, et al (2009) A 5’ promoter region SNP in NRG1 is associated with schizophrenia risk and type III isoform expression. Molecular Psychiatry 14: 741–743. 19626024 10.1038/mp.2008.150 PMC3271936

[39] Wood LS, Pickering EH, Dechairo BM (2007) Significant support for DAO as a schizophrenia susceptibility locus: examination of five genes putatively associated with schizophrenia. Biological Psychiatry 61: 1195–1199. 17055463 10.1016/j.biopsych.2006.07.005

[40] Opgen-Rhein C, Lencz T, Burdick KE, Neuhaus AH, DeRosse P, et al (2008) Genetic variation in the DAOA gene complex: impact on susceptibility for schizophrenia and on cognitive performance. Schizophrenia Research 103: 169–177. 18541412 10.1016/j.schres.2008.04.020 PMC2605318

[41] Hwu H-G, Liu C-M, Liu Y-L, Fann CS-J, Yang W-C, et al.. (2008) The polymorphisms in the promoter regions of NRG1 are associated with schizophrenia. The World Congress for Psychiatric Genetics, Osaka, Japan.

[42] Liu Y, Liu C, Fann C, Yang U, Yang W, et al.. (2012) Haplotypes of the D-amino Acid Oxidase Gene are Significantly Associated with Schizophrenia: A Systematic Sequencing Study. (Manuscript under review). American Journal of Psychiatry.

[43] Liu YL, Fann CS, Liu CM, Chang CC, Wu JY, et al (2006) No association of G72 and D-amino acid oxidase genes with schizophrenia. Schizophrenia Research 87: 15–20. 16842973 10.1016/j.schres.2006.06.020

[44] Lin C-L, Liu C-M, Liu Y-L, Fann CS-J, Chang C-C, et al.. (2012) The LMBRD1 is associated with schizophrenia with attention deficit. (Manuscript in preparation).

[45] Mothet JP, Parent AT, Wolosker H, Brady RO Jr, Linden DJ, et al (2000) D-serine is an endogenous ligand for the glycine site of the N-methyl-D-aspartate receptor. Proc Natl Acad Sci U S A 97: 4926–4931. 10781100 10.1073/pnas.97.9.4926 PMC18334

[46] Chumakov I, Blumenfeld M, Guerassimenko O, Cavarec L, Palicio M, et al (2002) Genetic and physiological data implicating the new human gene G72 and the gene for D-amino acid oxidase in schizophrenia. Proceedings of the National Academy of Sciences of the United States of America 99: 13675–13680. 12364586 10.1073/pnas.182412499 PMC129739

[47] Hashimoto K, Fukushima T, Shimizu E, Komatsu N, Watanabe H, et al (2003) Decreased serum levels of D-serine in patients with schizophrenia: evidence in support of the N-methyl-D-aspartate receptor hypofunction hypothesis of schizophrenia. Archives of General Psychiatry 60: 572–576. 12796220 10.1001/archpsyc.60.6.572

[48] Barros CS, Calabrese B, Chamero P, Roberts AJ, Korzus E, et al (2009) Impaired maturation of dendritic spines without disorganization of cortical cell layers in mice lacking NRG1/ErbB signaling in the central nervous system. Proc Natl Acad Sci U S A 106: 4507–4512. 19240213 10.1073/pnas.0900355106 PMC2657442

[49] Hattori T, Shimizu S, Koyama Y, Yamada K, Kuwahara R, et al (2010) DISC1 regulates cell-cell adhesion, cell-matrix adhesion and neurite outgrowth. Mol Psychiatry 15: 798–809. 10.1038/mp.2010.60 20479754

[50] Young-Pearse TL, Suth S, Luth ES, Sawa A, Selkoe DJ (2010) Biochemical and Functional Interaction of Disrupted-in-Schizophrenia 1 and Amyloid Precursor Protein Regulates Neuronal Migration during Mammalian Cortical Development. J Neurosci 30: 10431–10440. 20685985 10.1523/JNEUROSCI.1445-10.2010 PMC3018837

[51] Kifle L, Ortiz D, Shea TB (2009) Deprivation of folate and B12 increases neurodegeneration beyond that accompanying deprivation of either vitamin alone. Journal of Alzheimer’s Disease 16: 533–540. 10.3233/JAD-2009-1006 19276548

[52] Rutsch F, Gailus S, Suormala T, Fowler B (2010) LMBRD1: the gene for the cblF defect of vitamin B(12) metabolism. J Inherit Metab Dis. 10.1007/s10545-009-9032-7 20127417

[53] Zhang Z, Majava V, Greffier A, Hayes RL, Kursula P, et al (2009) Collapsin response mediator protein-2 is a calmodulin-binding protein. Cell Mol Life Sci 66: 526–536. 19151921 10.1007/s00018-008-8362-1 PMC4428678

[54] Martins-de-Souza D, Schmitt A, Roder R, Lebar M, Schneider-Axmann T, et al.. (2010) Sex-specific proteome differences in the anterior cingulate cortex of schizophrenia. J Psychiatr Res. 10.1016/j.jpsychires.2010.03.003 20381070

[55] Guo J, Meng F, Fu X, Song B, Yan X, et al (2004) N-methyl-D-aspartate receptor and L-type voltage-gated Ca2+ channel activation mediate proline-rich tyrosine kinase 2 phosphorylation during cerebral ischemia in rats. Neurosci Lett 355: 177–180. 14732460 10.1016/j.neulet.2003.10.076

[56] Harrison LM, LaHoste GJ (2006) Rhes, the Ras homolog enriched in striatum, is reduced under conditions of dopamine supersensitivity. Neuroscience 137: 483–492. 16352400 10.1016/j.neuroscience.2005.08.017

[57] Tselnicker I, Tsemakhovich VA, Dessauer CW, Dascal N (2010) Stargazin modulates neuronal voltage-dependent Ca(2+) channel Ca(v)2.2 by a Gbetagamma-dependent mechanism. J Biol Chem 285: 20462–20471. 20435886 10.1074/jbc.M110.121277 PMC2898357

[58] Sumioka A, Yan D, Tomita S (2010) TARP phosphorylation regulates synaptic AMPA receptors through lipid bilayers. Neuron 66: 755–767. 20547132 10.1016/j.neuron.2010.04.035 PMC2887694

[59] Lalonde JP, Lim R, Ingley E, Tilbrook PA, Thompson MJ, et al (2004) HLS5, a novel RBCC (ring finger, B box, coiled-coil) family member isolated from a hemopoietic lineage switch, is a candidate tumor suppressor. J Biol Chem 279: 8181–8189. 14662771 10.1074/jbc.M306751200

[60] Gottesman, II, Gould TD (2003) The endophenotype concept in psychiatry: etymology and strategic intentions. Am J Psychiatry 160: 636–645. 12668349 10.1176/appi.ajp.160.4.636

[61] Andreasen NC, Wilcox MA, Ho BC, Epping E, Ziebell S, et al.. (2011) Statistical epistasis and progressive brain change in schizophrenia: an approach for examining the relationships between multiple genes. Molecular Psychiatry. 10.1038/mp.2011.108 PMC3235542 21876540

[62] Beck LH, Bransome ED Jr, Mirsky AF, Rosvold HE, Sarason I (1956) A continuous performance test of brain damage. Journal of Consulting Psychology 20: 343–350. 13367264 10.1037/h0043220

[63] Faraone SV, Seidman LJ, Kremen WS, Toomey R, Pepple JR, et al (1999) Neuropsychological functioning among the nonpsychotic relatives of schizophrenic patients: a 4-year follow-up study. J Abnorm Psychol 108: 176–181. 10067004 10.1037//0021-843x.108.1.176

[64] Faraone SV, Kremen WS, Lyons MJ, Pepple JR, Seidman LJ, et al (1995) Diagnostic accuracy and linkage analysis: how useful are schizophrenia spectrum phenotypes? Am J Psychiatry 152: 1286–1290. 7653682 10.1176/ajp.152.9.1286

[65] Cornblatt BA, Keilp JG (1994) Impaired attention, genetics, and the pathophysiology of schizophrenia. Schizophrenia Bulletin 20: 31–46. 8197420 10.1093/schbul/20.1.31

[66] Chen WJ, Faraone SV (2000) Sustained attention deficits as markers of genetic susceptibility to schizophrenia. Am J Med Genet 97: 52–57. 10813804 10.1002/(sici)1096-8628(200021)97:1<52::aid-ajmg7>3.0.co;2-6

[67] Faraone SV, Seidman LJ, Kremen WS, Toomey R, Pepple JR, et al (2000) Neuropsychologic functioning among the nonpsychotic relatives of schizophrenic patients: the effect of genetic loading. Biol Psychiatry 48: 120–126. 10903408 10.1016/s0006-3223(99)00263-2

[68] Chen WJ, Hsiao CK, Hsiao LL, Hwu HG (1998) Performance of the Continuous Performance Test among community samples. Schizophrenia Bulletin 24: 163–174. 9502554 10.1093/oxfordjournals.schbul.a033308

[69] Chen WJ, Chang CH, Liu SK, Hwang TJ, Hwu HG (2004) Sustained attention deficits in nonpsychotic relatives of schizophrenic patients: a recurrence risk ratio analysis. Biological Psychiatry 55: 995–1000. 15121483 10.1016/j.biopsych.2004.01.010

[70] Hwu HG, Chen CH, Hwang TJ, Liu CM, Cheng JJ, et al (2002) Symptom patterns and subgrouping of schizophrenic patients: significance of negative symptoms assessed on admission. Schizophr Res 56: 105–119. 12084425 10.1016/s0920-9964(01)00251-1

[71] Pan WH, Fann CS, Wu JY, Hung YT, Ho MS, et al (2006) Han Chinese cell and genome bank in Taiwan: purpose, design and ethical considerations. Hum Hered 61: 27–30. 16534213 10.1159/000091834

[72] Hwu HG (1999) Psychiatric diagnostic assessment: Publication Committee, College of Medicine, National Taiwan University Taipei.

[73] Chen CH, Lee YR, Chung MY, Wei FC, Koong FJ, et al (1999) Systematic mutation analysis of the catechol O-methyltransferase gene as a candidate gene for schizophrenia. Am J Psychiatry 156: 1273–1275. 10450274 10.1176/ajp.156.8.1273

[74] Yuan HY, Chiou JJ, Tseng WH, Liu CH, Liu CK, et al (2006) FASTSNP: An always up-to-date and extendable service for SNP function analysis and prioritization. Nucleic Acids Research 34: W635–W641. 16845089 10.1093/nar/gkl236 PMC1538865

[75] Benjamini Y, Hochberg Y (1995) Controlling the false discovery rate: A practical and powerful approach to multiple testing. Journal of the Royal Statistical Society Series B-Methodological 57: 289–300.

[76] Guo SW, Thompson EA (1992) Performing the exact test of Hardy-Weinberg proportion for multiple alleles. Biometrics 48: 361–372. 1637966

[77] Servin B, Stephens M (2007) Imputation-based analysis of association studies: Candidate regions and quantitative traits. PLoS Genetics 3: 1296–1308. 10.1371/journal.pgen.0030114 PMC1934390 17676998

[78] SAS Publishing (2008) SAS/Genetics 9.2 User’s Guide. Cary, North Carolina. 256 p.

[79] Barrett JC, Fry B, Maller J, Daly MJ (2005) Haploview: Analysis and visualization of LD and haplotype maps. Bioinformatics 21: 263–265. 15297300 10.1093/bioinformatics/bth457

[80] Lewontin RC (1964) The interaction of selection and linkage. I. General considerations; heterotic models. Genetics 49: 49–67. 17248194 10.1093/genetics/49.1.49 PMC1210557

[81] Gabriel SB, Schaffner SF, Nguyen H, Moore JM, Roy J, et al (2002) The structure of haplotype blocks in the human genome. Science 296: 2225–2229. 12029063 10.1126/science.1069424

[82] Zhao JH, Curtis D, Sham PC (2000) Model-free analysis and permutation tests for allelic associations. Human Heredity 50: 133–139. 10799972 10.1159/000022901

[83] Yang H-C, Chen J-W, Liu C-M, Wen C-C, Liu Y-L, et al (2009) The Taiwan Schizophrenia Genetic Interaction Study. Genetic Epidemiology 33: 771–771.

[84] Ritchie MD, Hahn LW, Roodi N, Bailey LR, Dupont WD, et al (2001) Multifactor-dimensionality reduction reveals high-order interactions among estrogen-metabolism genes in sporadic breast cancer. American Journal of Human Genetics 69: 138–147. 11404819 10.1086/321276 PMC1226028

[85] Ritchie MD, Hahn LW, Moore JH (2003) Power of multifactor dimensionality reduction for detecting gene-gene interactions in the presence of genotyping error, missing data, phenocopy, and genetic heterogeneity. Genetic Epidemiology 24: 150–157. 12548676 10.1002/gepi.10218

[86] Hahn LW, Ritchie MD, Moore JH (2003) Multifactor dimensionality reduction software for detecting gene-gene and gene-environment interactions. Bioinformatics 19: 376–382. 12584123 10.1093/bioinformatics/btf869

[87] McClellan JM, Susser E, King MC (2007) Schizophrenia: A common disease caused by multiple rare alleles. British Journal of Psychiatry 190: 194–199. 17329737 10.1192/bjp.bp.106.025585

[88] Schell MJ (2004) The N-methyl D-aspartate receptor glycine site and D-serine metabolism: an evolutionary perspective. Philos Trans R Soc Lond B Biol Sci 359: 943–964. 15306409 10.1098/rstb.2003.1399 PMC1693380

[89] Verrall L, Burnet PW, Betts JF, Harrison PJ (2010) The neurobiology of D-amino acid oxidase and its involvement in schizophrenia. Molecular Psychiatry 15: 122–137. 19786963 10.1038/mp.2009.99 PMC2811712

[90] Shleper M, Kartvelishvily E, Wolosker H (2005) D-serine is the dominant endogenous coagonist for NMDA receptor neurotoxicity in organotypic hippocampal slices. J Neurosci 25: 9413–9417. 16221850 10.1523/JNEUROSCI.3190-05.2005 PMC6725696

[91] Gong XQ, Zabek RL, Bai D (2007) D-Serine inhibits AMPA receptor-mediated current in rat hippocampal neurons. Can J Physiol Pharmacol 85: 546–555. 17632590 10.1139/y07-040

[92] Bartos JA, Ulrich JD, Li H, Beazely MA, Chen Y, et al (2010) Postsynaptic clustering and activation of Pyk2 by PSD-95. The Journal of neuroscience: the official journal of the Society for Neuroscience 30: 449–463. 20071509 10.1523/JNEUROSCI.4992-08.2010 PMC2822408

[93] Xu J, Kurup P, Bartos JA, Patriarchi T, Hell JW, et al (2012) Striatal-enriched protein-tyrosine phosphatase (STEP) regulates Pyk2 kinase activity. The Journal of biological chemistry 287: 20942–20956. 22544749 10.1074/jbc.M112.368654 PMC3375518

[94] Ducharme G, Lowe GC, Goutagny R, Williams S (2012) Early alterations in hippocampal circuitry and theta rhythm generation in a mouse model of prenatal infection: implications for schizophrenia. PloS one 7. 10.1371/journal.pone.0029754 PMC3253085 22238649

[95] Morita Y, Ujike H, Tanaka Y, Otani K, Kishimoto M, et al (2007) A genetic variant of the serine racemase gene is associated with schizophrenia. Biol Psychiatry 61: 1200–1203. 17067558 10.1016/j.biopsych.2006.07.025

[96] Boks MPM, Rietkerk T, van de Beek MH, Sommer IE, de Koning TJ, et al (2007) Reviewing the role of the genes G72 and DAAO in glutamate neurotransmission in schizophrenia. European neuropsychopharmacology: the journal of the European College of Neuropsychopharmacology 17: 567–572. 17250995 10.1016/j.euroneuro.2006.12.003

[97] Ma TM, Abazyan S, Abazyan B, Nomura J, Yang C, et al.. (2012) Pathogenic disruption of DISC1-serine racemase binding elicits schizophrenia-like behavior via D-serine depletion. Molecular psychiatry. 10.1038/mp.2012.97 PMC3475769 22801410

[98] Kawazoe T, Tsuge H, Pilone MS, Fukui K (2006) Crystal structure of human D-amino acid oxidase: context-dependent variability of the backbone conformation of the VAAGL hydrophobic stretch located at the si-face of the flavin ring. Protein science: a publication of the Protein Society 15: 2708–2717. 17088322 10.1110/ps.062421606 PMC2242440

[99] Veitia RA (2010) A generalized model of gene dosage and dominant negative effects in macromolecular complexes. FASEB journal: official publication of the Federation of American Societies for Experimental Biology 24: 994–1002. 20007508 10.1096/fj.09-146969

[100] Chen L, Chetkovich DM, Petralia RS, Sweeney NT, Kawasaki Y, et al (2000) Stargazin regulates synaptic targeting of AMPA receptors by two distinct mechanisms. Nature 408: 936–943. 11140673 10.1038/35050030

[101] Bjarnadottir M, Misner DL, Haverfield-Gross S, Bruun S, Helgason VG, et al (2007) Neuregulin1 (NRG1) signaling through Fyn modulates NMDA receptor phosphorylation: differential synaptic function in NRG1+/− knock-outs compared with wild-type mice. The Journal of neuroscience: the official journal of the Society for Neuroscience 27: 4519–4529. 17460065 10.1523/JNEUROSCI.4314-06.2007 PMC6672983

[102] Guarente L (1993) Synthetic enhancement in gene interaction: a genetic tool come of age. Trends in Genetics 9: 362–366. 8273152 10.1016/0168-9525(93)90042-g

[103] Brunelin J, Fecteau S, Suaud-Chagny M-F (2012) Abnormal Striatal Dopamine Transmission in Schizophrenia. Current medicinal chemistry. 10.2174/0929867311320030011 PMC3866953 23157632

[104] Errico F, Santini E, Migliarini S, Borgkvist A, Centonze D, et al (2008) The GTP-binding protein Rhes modulates dopamine signalling in striatal medium spiny neurons. Molecular and cellular neurosciences 37: 335–345. 18035555 10.1016/j.mcn.2007.10.007

[105] Tan HY, Chen AG, Kolachana B, Apud JA, Mattay VS, et al (2012) Effective connectivity of AKT1-mediated dopaminergic working memory networks and pharmacogenetics of anti-dopaminergic treatment. Brain: a journal of neurology 135: 1436–1445. 22525159 10.1093/brain/aws068 PMC3338927

[106] Bang S, Steenstra C, Kim SF (2012) Striatum specific protein, Rhes regulates AKT pathway. Neuroscience Letters 521: 142–147. 22683505 10.1016/j.neulet.2012.05.073 PMC3389258

[107] Howes OD, Kapur S (2009) The dopamine hypothesis of schizophrenia: version III–the final common pathway. Schizophrenia bulletin 35: 549–562. 19325164 10.1093/schbul/sbp006 PMC2669582

[108] Moghaddam B, Javitt D (2012) From revolution to evolution: the glutamate hypothesis of schizophrenia and its implication for treatment. Neuropsychopharmacology: official publication of the American College of Neuropsychopharmacology 37: 4–15. 21956446 10.1038/npp.2011.181 PMC3238069

[109] Hosmer DW, Lemeshow S (2000) Applied Logistic Regression, Second Edition. New York: John Wiley & Sons.

