# Supplementary figures and images for: The DAO Gene Is Associated with Schizophrenia and Interacts with Other Genes in the Taiwan Han Chinese Population
Source: PLoS One. 2013 Mar 28;8(3):e60099. doi: 10.1371/journal.pone.0060099 (PMC3610748; doi:10.1371/journal.pone.0060099)

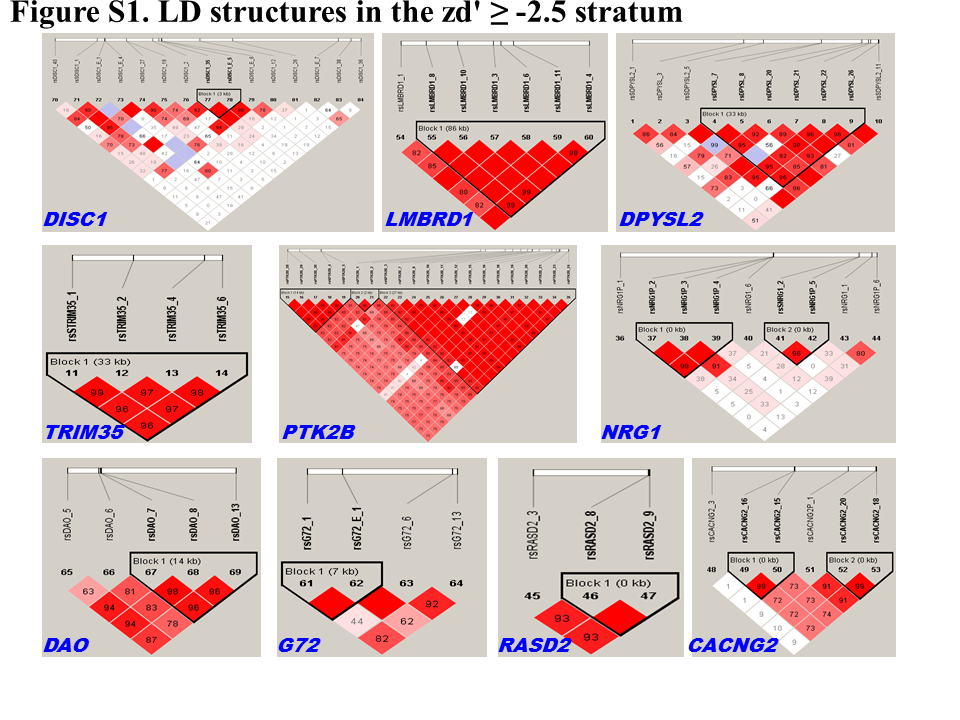

Supplement: Figure S1 — The LD structures of 84 SNPs in 10 candidate genes for the Zd’ ≥ −2.5 stratum. For each gene, the id of each SNP in the gene is listed, and the locations reflect the relative physical positions of the SNPs (in units of base pairs). The LD coefficient, D’, is provided unless D’ = 1. The color scheme for D’ presentation is as follows: white depicts the case of D’ <1 and LOD <2; blue depicts the case of D’ = 1 and LOD <2; pink or light red depicts the case of D’ <1 and LOD ≥2; bright red depicts the case of D’ = 1 and LOD ≥2. The LD block(s) within each gene is marked by an inverted triangle based on Gabriel’s method [81]. (TIF) [file pone.0060099.s001.tif]

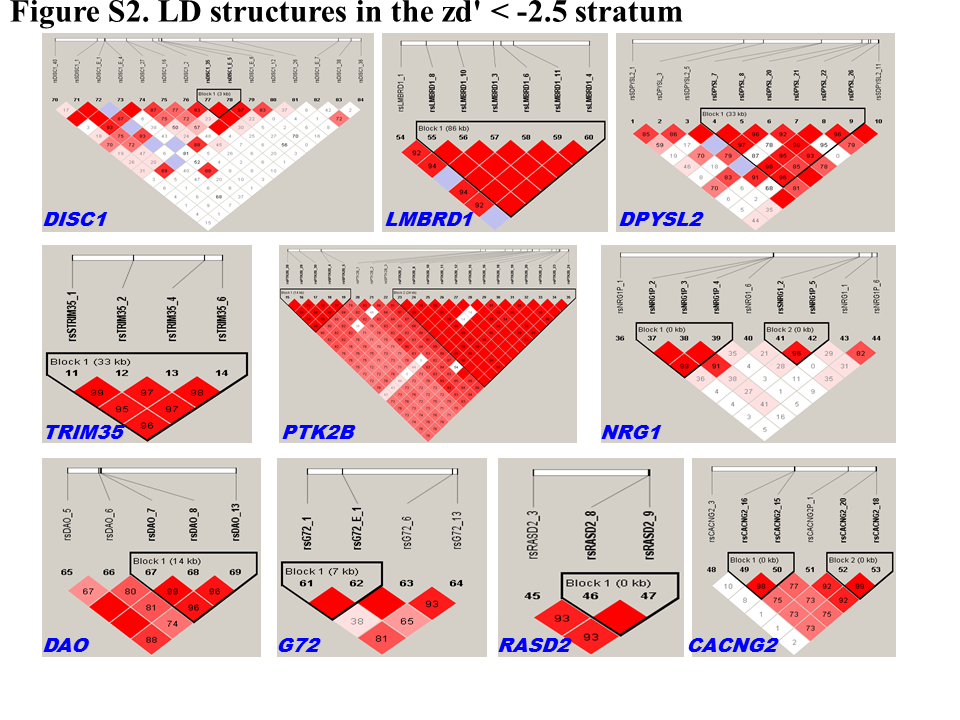

Supplement: Figure S2 — The LD structures of 84 SNPs in 10 candidate genes for the Zd’<−2.5 stratum. For each gene, the id of each SNP in the gene is listed, and the locations reflect the relative physical positions of the SNPs (in units of base pairs). The LD coefficient, D’, is provided unless D’ = 1. The color scheme for D’ presentation is as follows: white depicts the case of D’ <1 and LOD <2; blue depicts the case of D’ = 1 and LOD <2; pink or light red depicts the case of D’ <1 and LOD ≥2; bright red depicts the case of D’ = 1 and LOD ≥2. The LD block(s) within each gene is marked by an inverted triangle based on Gabriel’s method [81]. (TIF) [file pone.0060099.s002.tif]

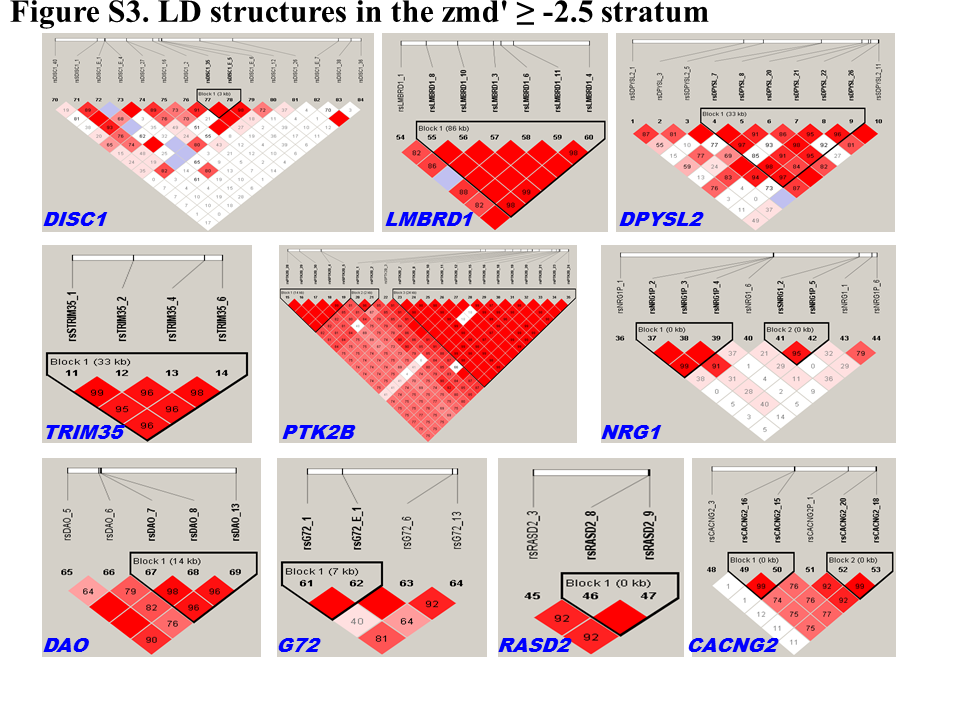

Supplement: Figure S3 — The LD structures of 84 SNPs in 10 candidate genes for the Zmd’ ≥ −2.5 stratum. For each gene, the id of each SNP in the gene is listed, and the locations reflect the relative physical positions of the SNPs (in units of base pairs). The LD coefficient, D’, is provided unless D’ = 1. The color scheme for D’ presentation is as follows: white depicts the case of D’ <1 and LOD <2; blue depicts the case of D’ = 1 and LOD <2; pink or light red depicts the case of D’ <1 and LOD ≥2; bright red depicts the case of D’ = 1 and LOD ≥2. The LD block(s) within each gene is marked by an inverted triangle based on Gabriel’s method [81]. (TIF) [file pone.0060099.s003.tif]

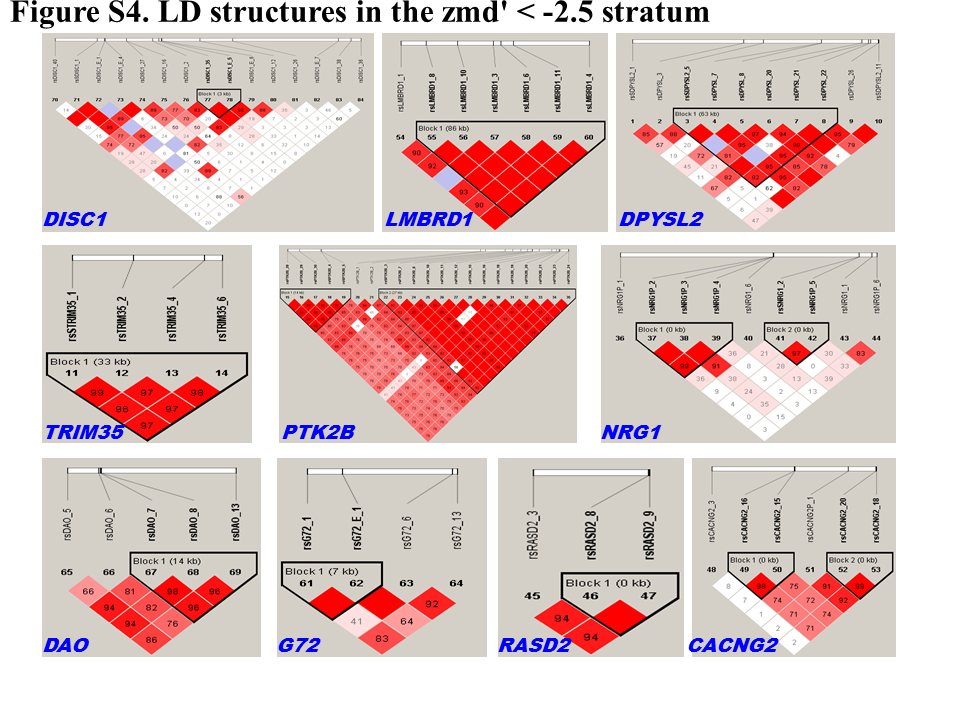

Supplement: Figure S4 — The LD structures of 84 SNPs in 10 candidate genes for the Zmd’<−2.5 stratum. For each gene, the id of each SNP in the gene is listed, and the locations reflect the relative physical positions of the SNPs (in units of base pairs). The LD coefficient, D’, is provided unless D’ = 1. The color scheme for D’ presentation is as follows: white depicts the case of D’ <1 and LOD <2; blue depicts the case of D’ = 1 and LOD <2; pink or light red depicts the case of D’ <1 and LOD ≥2; bright red depicts the case of D’ = 1 and LOD ≥2. The LD block(s) within each gene is marked by an inverted triangle based on Gabriel’s method [81]. (TIF) [file pone.0060099.s004.tif]
