# Supplementary material for: The DAO Gene Is Associated with Schizophrenia and Interacts with Other Genes in the Taiwan Han Chinese Population
Source: PLoS One. 2013 Mar 28;8(3):e60099. doi: 10.1371/journal.pone.0060099 (PMC3610748; doi:10.1371/journal.pone.0060099)
Supplement: Table S1 — Results of single-locus association tests. Chromosome, gene name, SNP ID, rs number, region, function, and physical position are shown. The raw p-values and FDR-adjusted p-values are shown for genotype-based (X_G), allele-based (X_A), and trend-based (X_T) association tests in an unstratified analysis of overall samples and a stratified analysis of four CPT strata, Zd’ ≥ −2.5, Zd’<−2.5, Zmd’ ≥ −2.5, and Zmd’<−2.5. (PDF) [file pone.0060099.s005.pdf]

Table S1. Results of single locus association tests

								Raw p-values															FDR adjusted p-values														
SNP information								Overall			zd' ≥ -2.5			zd' < -2.5			zmd' ≥ -2.5			zmd' < -2.5			Overall			zd' ≥ -2.5			zd' < -2.5			zmd' ≥ -2.5			zmd' < -2.5		
obs	chr	gene	SNPID	rsSNPID	Region	Function	position	X_G	X_A	X_T	X_G	X_A	X_T	X_G	X_A	X_T	X_G	X_A	X_T	X_G	X_A	X_T	X_G	X_A	X_T	X_G	X_A	X_T	X_G	X_A	X_T	X_G	X_A	X_T	X_G	X_A	X_T
1	1q	DISC1	rsDISC1_40	rs1865225	intron 1	intron	229829627	0.5676	0.4284	0.4306	0.0949	0.0385	0.0372	0.5217	0.3628	0.3740	0.0825	0.0288	0.0256	0.4979	0.6625	0.6554	0.8786	0.8935	0.8912	0.6923	0.5213	0.5096	0.9572	0.9831	0.9478	0.6370	0.4018	0.3776	0.9683	1.0000	1.0000
2	1q	DISC1	rsSDISC1_1	rs28434312	intron 1	intron	229886536	0.1025	0.1860	0.1846	0.3823	1.0000	1.0000	0.0431	0.0564	0.0476	0.5201	0.6452	0.6500	0.0294	0.0497	0.0485	0.8208	0.8935	0.8912	0.7832	1.0000	1.0000	0.3635	0.5731	0.5820	0.7811	0.8794	0.8721	0.6014	0.8350	0.8148
3	1q	DISC1	rsDISC1P_3	rs12034296	intron 1	intron	229886700	-	-	-	-	-	-	-	-	-	-	-	-	-	-	-	-	-	-	-	-	-	-	-	-	-	-	-	-	-	-
4	1q	DISC1	rsDISC1_E_1	rs56020408	exon 2	missense	229896474	0.3028	0.2964	0.3028	0.4392	0.4422	0.4392	0.2699	0.2672	0.2699	0.4042	0.4044	0.4042	0.4144	0.4216	0.4144	0.8786	0.8935	0.8912	0.8385	0.8254	0.8198	0.8733	0.8633	0.8720	0.7634	0.7467	0.7354	0.9683	1.0000	1.0000
5	1q	DISC1	rsDISC1_23	rs3738401	exon2	missense	229896918	-	-	-	-	-	-	-	-	-	-	-	-	-	-	-	-	-	-	-	-	-	-	-	-	-	-	-	-	-	-
6	1q	DISC1	rsDISC1_E_4	rs55795950	exon 2	missense	229897110	0.8625	0.8645	0.8625	0.5196	0.5240	0.5196	1.0000	1.0000	1.0000	0.8316	0.8333	0.8316	0.3693	0.3697	0.3693	0.9660	0.9732	0.9743	0.8579	0.8638	0.8660	1.0000	1.0000	1.0000	0.9191	0.9338	0.9310	0.9124	1.0000	1.0000
7	1q	DISC1	rsDISC1_27	rs11122324	intron 3	intron	229925804	0.0115	0.1140	0.1163	0.1038	0.7028	0.7071	0.0074	0.0503	0.0495	0.3035	0.6889	0.6818	0.0018	0.0249	0.0208	0.4830	0.8935	0.8912	0.6923	0.8638	0.8660	0.2072	0.5731	0.5820	0.7634	0.8875	0.8861	0.1120	0.8350	0.8148
8	1q	DISC1	rsDISC1_16	rs12023889	intron 3	intron	229926137	0.6085	0.3840	0.3784	0.3164	0.2008	0.1924	0.3539	0.4937	0.4639	0.6154	0.4178	0.4115	0.4379	0.2266	0.2085	0.8786	0.8935	0.8912	0.7782	0.7157	0.7009	0.8743	1.0000	0.9987	0.8077	0.7467	0.7354	0.9683	0.9496	0.9218
9	1q	DISC1	rsDISC1_2	rs2793091	intron 4	intron	229961231	0.0607	0.2856	0.2855	0.2509	0.9621	0.9661	0.0224	0.1014	0.0950	0.5693	0.8881	0.8836	0.0040	0.1010	0.1003	0.8208	0.8935	0.8912	0.7305	0.9767	0.9777	0.3635	0.6552	0.5820	0.8077	0.9564	0.9516	0.1120	0.8904	0.8851
10	1q	DISC1	rsDISC1_35	rs3738402	exon 5	cds-synon	229969633	0.2938	0.4033	0.4112	0.1126	0.4839	0.4862	0.4531	0.4003	0.3919	0.1365	0.7235	0.7328	0.2431	0.1794	0.1736	0.8786	0.8935	0.8912	0.6923	0.8613	0.8617	0.8851	0.9879	0.9478	0.6370	0.8875	0.8861	0.7850	0.9496	0.9218
11	1q	DISC1	rsDISC1_E_5	rs2492367	exon 6	cds-synon	229973212	0.8436	0.8017	0.8024	0.8640	0.6316	0.6356	0.2203	0.7421	0.7359	0.4745	0.2966	0.3182	0.2309	0.9484	0.9522	0.9660	0.9485	0.9493	0.9383	0.8638	0.8660	0.8733	1.0000	0.9987	0.7665	0.6734	0.7034	0.7850	1.0000	1.0000
12	1q	DISC1	rsDISC1_E_6	rs56229136	exon 6	missense	229973396	0.3777	0.2041	0.2113	0.4826	0.5830	0.5873	0.2086	0.0833	0.0873	0.2621	0.2260	0.2470	0.5184	0.2255	0.2468	0.8786	0.8935	0.8912	0.8579	0.8638	0.8660	0.8733	0.6552	0.5820	0.7634	0.6259	0.6287	0.9683	0.9496	0.9324
13	1q	DISC1	rsDISC1_12	rs56071042	intron 9	intron	230024766	0.5092	0.2859	0.2913	0.2696	0.2662	0.2702	0.9483	1.0000	1.0000	0.5300	0.3618	0.3635	0.8957	0.8766	0.8799	0.8786	0.8935	0.8912	0.7305	0.7157	0.7009	1.0000	1.0000	1.0000	0.7811	0.7068	0.7113	0.9773	1.0000	1.0000
14	1q	DISC1	rsDISC1_26	rs3082	intron 9	intron	230069015	0.9562	0.9696	0.9675	0.7793	0.8525	0.8566	0.8766	0.6337	0.6455	0.6065	0.7284	0.7286	0.9697	0.8760	0.8868	0.9775	0.9816	0.9816	0.9383	0.9528	0.9468	1.0000	1.0000	0.9987	0.8077	0.8875	0.8861	0.9773	1.0000	1.0000
15	1q	DISC1	rsDISC1_E_7	rs821616	exon 11	missense	230211221	0.1128	0.1683	0.1631	0.1290	0.6619	0.6634	0.1643	0.0902	0.0813	0.6110	0.5892	0.5674	0.0721	0.3224	0.3202	0.8208	0.8935	0.8912	0.6923	0.8638	0.8660	0.8013	0.6552	0.5820	0.8077	0.8704	0.8511	0.7571	0.9672	0.9618
16	1q	DISC1	rsDISC1_38	rs16856254	intron 11	intron	230228487	0.2972	0.4122	0.4046	0.3613	0.2139	0.2204	0.2822	0.6795	0.6768	0.4489	0.3306	0.3362	0.5541	0.5728	0.5630	0.8786	0.8935	0.8912	0.7782	0.7157	0.7009	0.8733	1.0000	0.9987	0.7634	0.7068	0.7060	0.9683	1.0000	1.0000
17	1q	DISC1	rsDISC1_36	rs41303123	exon 13	3' UTR	230241611	0.6555	0.4141	0.4158	0.7883	0.5372	0.5498	0.8497	0.5885	0.5765	0.6290	0.3570	0.3641	0.9480	0.8991	0.8955	0.8881	0.8935	0.8912	0.9383	0.8638	0.8660	1.0000	1.0000	0.9987	0.8129	0.7068	0.7113	0.9773	1.0000	1.0000
18	6q	LMBRD1	rsLMBRD1_1	rs9354878	intron 14	intron	70450792	0.7130	0.4225	0.4166	0.6278	0.3903	0.3905	0.9567	0.9161	0.9101	0.5963	0.3103	0.3103	0.8156	0.6769	0.6796	0.9092	0.8935	0.8912	0.9383	0.7480	0.7646	1.0000	1.0000	1.0000	0.8077	0.6859	0.7034	0.9773	1.0000	1.0000
19	6q	LMBRD1	rsLMBRD1_8	rs9354880	exon14	missense	70464186	0.3408	0.4807	0.4987	0.1960	0.2783	0.2800	0.0445	0.0330	0.0350	0.3815	0.6918	0.7044	0.2710	0.2951	0.2976	0.8786	0.8935	0.8912	0.6923	0.7157	0.7009	0.3635	0.5731	0.5820	0.7634	0.8875	0.8861	0.7850	0.9672	0.9618
20	6q	LMBRD1	rsLMBRD1_9	rs17854411	exon12	missense	70467383	-	-	-	-	-	-	-	-	-	-	-	-	-	-	-	-	-	-	-	-	-	-	-	-	-	-	-	-	-	-
21	6q	LMBRD1	rsLMBRD1_7	rs34327883	exon 9	cds-synon	70480372	-	-	-	-	-	-	-	-	-	-	-	-	-	-	-	-	-	-	-	-	-	-	-	-	-	-	-	-	-	-
22	6q	LMBRD1	rsLMBRD1_10	rs3799112	intron 8	intron	70481020	0.0961	0.1969	0.2008	0.2080	0.6085	0.6057	0.0043	0.0058	0.0059	0.1442	0.8413	0.8351	0.0503	0.0448	0.0451	0.8208	0.8935	0.8912	0.6989	0.8638	0.8660	0.1806	0.2436	0.2478	0.6375	0.9338	0.9310	0.6036	0.8350	0.8148
23	6q	LMBRD1	rsLMBRD1_3	rs16868111	intron 7	intron	70500149	0.7705	0.7060	0.7153	0.3556	0.3918	0.3951	0.2473	0.1346	0.1406	0.4255	0.7673	0.7701	0.5104	0.3475	0.3496	0.9116	0.9008	0.8987	0.7782	0.7480	0.7646	0.8733	0.7067	0.7382	0.7634	0.8875	0.8861	0.9683	1.0000	1.0000
24	6q	LMBRD1	rsLMBRD1_6	rs9346341	intron 7	intron	70504033	0.2354	0.2460	0.2309	0.3973	0.9170	0.9185	0.1455	0.1127	0.0960	0.3847	0.9107	0.9117	0.2082	0.2068	0.1898	0.8786	0.8935	0.8912	0.7946	0.9767	0.9777	0.8013	0.6762	0.5820	0.7634	0.9672	0.9665	0.7850	0.9496	0.9218
25	6q	LMBRD1	rsLMBRD1_11	rs6904680	intron 5	intron	70510570	0.3253	0.4914	0.4796	0.1941	0.2520	0.2553	0.0445	0.0359	0.0398	0.3442	0.6666	0.6541	0.2626	0.3156	0.3158	0.8786	0.8935	0.8912	0.6923	0.7157	0.7009	0.3635	0.5731	0.5820	0.7634	0.8875	0.8721	0.7850	0.9672	0.9618
26	6q	LMBRD1	rsLMBRD1_5	rs12214456	exon 5	missense	70515997	-	-	-	-	-	-	-	-	-	-	-	-	-	-	-	-	-	-	-	-	-	-	-	-	-	-	-	-	-	-
27	6q	LMBRD1	rsLMBRD1_4	rs9454892	intron 2	intron	70550934	0.2670	0.6401	0.6573	0.2670	0.3571	0.3610	0.0476	0.0614	0.0693	0.4234	0.7636	0.7680	0.1143	0.2488	0.2618	0.8786	0.8947	0.8941	0.7305	0.7480	0.7646	0.3635	0.5731	0.5820	0.7634	0.8875	0.8861	0.7850	0.9500	0.9324
28	6q	LMBRD1	rsLMBRD1_2	rs2305840	intron 1	intron	70557239	-	-	-	-	-	-	-	-	-	-	-	-	-	-	-	-	-	-	-	-	-	-	-	-	-	-	-	-	-	-
29	8p	DPYSL2	rsSDPYSL2_1	rs4872449	5' near gene	5' near gene	26426431	0.3841	0.2475	0.2426	0.1893	0.0711	0.0748	0.6707	0.4170	0.4019	0.0888	0.0333	0.0326	0.9541	0.9206	0.9166	0.8786	0.8935	0.8912	0.6923	0.5213	0.5096	0.9666	0.9879	0.9478	0.6370	0.4018	0.3776	0.9773	1.0000	1.0000
30	8p	DPYSL2	rsDPYSL_3	rs379266	intron 1	intron	26492026	0.9748	0.7983	0.8008	0.8847	0.8368	0.8431	0.9328	0.7250	0.7269	0.6906	0.4056	0.4024	0.3570	0.1845	0.1827	0.9775	0.9485	0.9493	0.9383	0.9528	0.9468	1.0000	1.0000	0.9987	0.8407	0.7467	0.7354	0.9124	0.9496	0.9218
31	8p	DPYSL2	rsDPYSL_4	rs408753	intron 3	intron	26497479	-	-	-	-	-	-	-	-	-	-	-	-	-	-	-	-	-	-	-	-	-	-	-	-	-	-	-	-	-	-
32	8p	DPYSL2	rsSDPYSL2_5	rs62491913	intron 3	intron	26503707	0.5987	0.6283	0.6372	0.4192	0.3856	0.4005	0.6108	0.9137	0.9110	0.3908	0.7484	0.7535	0.4816	0.2714	0.2664	0.8786	0.8947	0.8941	0.8189	0.7480	0.7646	0.9666	1.0000	1.0000	0.7634	0.8875	0.8861	0.9683	0.9672	0.9324
33	8p	DPYSL2	rsDPYSL_6	rs74972796	intron 3	intron	26533093	-	-	-	-	-	-	-	-	-	-	-	-	-	-	-	-	-	-	-	-	-	-	-	-	-	-	-	-	-	-
34	8p	DPYSL2	rsDPYSL_7	rs2289593	exon 4	missense	26537614	0.9345	0.7996	0.7905	0.5764	0.3502	0.3414	0.9148	1.0000	1.0000	0.8917	0.6270	0.6176	0.9210	0.7523	0.7460	0.9775	0.9485	0.9493	0.8966	0.7480	0.7646	1.0000	1.0000	1.0000	0.9481	0.8794	0.8721	0.9773	1.0000	1.0000
35	8p	DPYSL2	rsDPYSL_8	rs327222	exon 4	cds-synon	26537688	0.5418	0.5425	0.5272	0.6966	0.9453	0.9434	0.5121	0.4140	0.4062	0.7888	0.9409	0.9376	0.3594	0.2832	0.2633	0.8786	0.8935	0.8912	0.9383	0.9767	0.9777	0.9572	0.9879	0.9478	0.9055	0.9757	0.9723	0.9124	0.9672	0.9324
36	8p	DPYSL2	rsDPYSL_15	rs28470992	intron 8	intron	26556345	-	-	-	-	-	-	-	-	-	-	-	-	-	-	-	-	-	-	-	-	-	-	-	-	-	-	-	-	-	-
37	8p	DPYSL2	rsDPYSL_20	rs9644117	intron 11	intron	26561435	0.9493	1.0000	1.0000	0.8954	0.6721	0.6774	0.8557	0.6038	0.6088	0.9679	0.8449	0.8423	0.8694	0.7720	0.7732	0.9775	1.0000	1.0000	0.9383	0.8638	0.8660	1.0000	1.0000	0.9987	0.9808	0.9338	0.9310	0.9773	1.0000	1.0000
38	8p	DPYSL2	rsDPYSL_21	rs55685919	intron 11	intron	26565540	0.7577	0.6817	0.6818	0.7924	0.7035	0.7045	1.0000	1.0000	1.0000	0.6489	0.6010	0.6078	0.8484	0.7635	0.7559	0.9092	0.8947	0.8949	0.9383	0.8638	0.8660	1.0000	1.0000	1.0000	0.8257	0.8704	0.8721	0.9773	1.0000	1.0000
39	8p	DPYSL2	rsDPYSL_22	rs708621	exon 13	cds-synon	26566709	0.9766	0.8744	0.8760	0.9484	0.9300	0.9269	0.5692	0.5796	0.5685	0.9691	1.0000	1.0000	0.8350	0.5646	0.5767	0.9775	0.9732	0.9743	0.9598	0.9767	0.9777	0.9666	1.0000	0.9987	0.9808	1.0000	1.0000	0.9773	1.0000	1.0000
40	8p	DPYSL2	rsDPYSL_23	rs11863	exon 14	3' UTR	26570233	-	-	-	-	-	-	-	-	-	-	-	-	-	-	-	-	-	-	-	-	-	-	-	-	-	-	-	-	-	-
41	8p	DPYSL2	rsDPYSL_26	rs45471201	exon 14	3' UTR	26571055	0.6295	0.5308	0.5328	0.2654	0.3556	0.3542	0.9332	1.0000	1.0000	0.7284	0.6491	0.6393	0.6921	0.6554	0.6494	0.8786	0.8935	0.8912	0.7305	0.7480	0.7646	1.0000	1.0000	1.0000	0.8741	0.8794	0.8721	0.9773	1.0000	1.0000
42	8p	DPYSL2	rsSDPYSL2_11	rs17666	exon 14	3' UTR	26571375	0.9422	0.7185	0.7218	0.5383	0.3873	0.3887	0.8494	0.7446	0.7518	0.5933	0.3417	0.3494	0.3455	0.8239	0.8273	0.9775	0.9008	0.8987	0.8579	0.7480	0.7646	1.0000	1.0000	0.9987	0.8077	0.7068	0.7113	0.9124	1.0000	1.0000
43	8p	TRIM35	rsSTRIM35_1	rs957008	IGR	3' downstrea	27190017	0.5863	0.3094	0.3224	0.1573	0.0684	0.0667	0.8824	0.6308	0.6257	0.1178	0.0452	0.0463	0.8892	0.6430	0.6499	0.8786	0.8935	0.8912	0.6923	0.5213	0.5096	1.0000	1.0000	0.9987	0.6370	0.4018	0.3776	0.9773	1.0000	1.0000
44	8p	TRIM35	rsTRIM35_2	rs12386854	intron 3	intron	27203856	0.3533	0.2414	0.2399	0.1050	0.0487	0.0533	0.5870	0.4675	0.4661	0.0620	0.0329	0.0357	0.7166	0.4902	0.4915	0.8786	0.8935	0.8912	0.6923	0.5213	0.5096	0.9666	1.0000	0.9987	0.6370	0.4018	0.3776	0.9773	1.0000	1.0000
45	8p	TRIM35	rsTRIM35_4	rs2322604	intron 1	intron	27220063	0.4110	0.3649	0.3743	0.1424	0.0666	0.0708	0.6043	0.8433	0.8492	0.1337	0.0985	0.0914	0.9154	0.9544	0.9533	0.8786	0.8935	0.8912	0.6923	0.5213	0.5096	0.9666	1.0000	1.0000	0.6370	0.4137	0.3839	0.9773	1.0000	1.0000
46	8p	TRIM35	rsTRIM35_5	rs2322605	intron 1	intron	27220366	-	-	-	-	-	-	-	-	-	-	-	-	-	-	-	-	-	-	-	-	-	-	-	-	-	-	-	-	-	-
47	8p	TRIM35	rsTRIM35_6	rs13253058	intron 1	intron	27223947	0.5192	0.4119	0.4169	0.1632	0.0722	0.0818	0.6834	0.8974	0.8985	0.2041	0.1035	0.1121	0.8701	0.9048	0.9078	0.8786	0.8935	0.8912	0.6923	0.5213	0.5096	0.9666	1.0000	1.0000	0.7454	0.4140	0.4448	0.9773	1.0000	1.0000
48	8p	PTK2B	rsPTK2B_28	rs1879187	intron 5	intron	27249483	0.1368	0.6695	0.6660	0.1712	0.2950	0.2792	0.4404	0.6748	0.6689	0.0597	0.0756	0.0713	0.1683	0.9211	0.9235	0.8208	0.8947	0.8941	0.6923	0.7157	0.7009	0.8820	1.0000	0.9987	0.6370	0.4018	0.3776	0.7850	1.0000	1.0000
49	8p	PTK2B	rsPTK2B_29	rs1879188	intron 5	intron	27249840	0.1358	0.6762	0.6559	0.1680	0.2982	0.2837	0.4410	0.6777	0.6709	0.0542	0.0790	0.0653	0.1765	0.9238	0.9219	0.8208	0.8947	0.8941	0.6923	0.7157	0.7009	0.8820	1.0000	0.9987	0.6370	0.4018	0.3776	0.7850	1.0000	1.0000
50	8p	PTK2B	rsPTK2B_30	rs1879189	intron 5	intron	27254801	0.1210	0.5949	0.5794	0.1450	0.2893	0.2775	0.3885	0.6003	0.5901	0.0373	0.0659	0.0552	0.1782	1.0000	1.0000	0.8208	0.8947	0.8941	0.6923	0.7157	0.7009	0.8820	1.0000	0.9987	0.6370	0.4018	0.3776	0.7850	1.0000	1.0000
51	8p	PTK2B	rsSPTK2B_4	rs6987305	intron 5	intron	27264043	0.3961	0.2273	0.2290	0.1633	0.0721	0.0726	0.5242	0.2546	0.2625	0.1021	0.0503	0.0542	0.6150	0.3719	0.3645	0.8786	0.8935	0.8912	0.6923	0.5213	0.5096	0.9572	0.8555	0.8720	0.6370	0.4018	0.3776	0.9683	1.0000	1.0000
52	8p	PTK2B	rsSPTK2B_5	rs6987465	intron 5	intron	27264179	0.1120	0.6066	0.6082	0.1502	0.2964	0.2721	0.4122	0.6079	0.5826	0.0426	0.0861	0.0650	0.2137	1.0000	1.0000	0.8208	0.8947	0.8941	0.6923	0.7157	0.7009	0.8820	1.0000	0.9987	0.6370	0.4018	0.3776	0.7850	1.0000	1.0000
53	8p	PTK2B	rsPTK2B_1	rs1030526	exon 7	cds-synon	27333454	0.1026	0.1338	0.1310	0.1608	0.0940	0.0850	0.0616	0.2206	0.2257	0.1549	0.0918	0.0854	0.0439	0.1952	0.2004	0.8208	0.8935	0.8912	0.6923	0.5213	0.5096	0.3980	0.8555	0.8720	0.6506	0.4059	0.3776	0.6036	0.9496	0.9218
54	8p	PTK2B	rsPTK2B_2	rs2241652	intron 8	intron	27335622	0.2538	0.1171	0.1048	0.3334	0.1654	0.1411	0.3426	0.2227	0.2002	0.0701	0.0470	0.0353	0.8695	0.6729	0.6487	0.8786	0.8935	0.8912	0.7782	0.7157	0.6972	0.8743	0.8555	0.8720	0.6370	0.4018	0.3776	0.9773	1.0000	1.0000
55	8p	PTK2B	rsSPTK2B_3	rs56373327	inron 11	intron	27344231	0.9178	0.9329	0.9297	0.9139	0.9162	0.9139	0.2864	0.3298	0.3222	0.3891	0.4146	0.3891	0.5246	1.0000	1.0000	0.9775	0.9732	0.9743	0.9383	0.9767	0.9777	0.8733	0.9234	0.9195	0.7634	0.7467	0.7354	0.9683	1.0000	1.0000
56	8p	PTK2B	rsPTK2B_7	rs919493	intron 15	intron	27347474	0.4843	0.4702	0.4654	0.8475	0.6110	0.6208	0.7480	0.6474	0.6463	0.3845	0.1902	0.1863	0.5925	0.7701	0.7731	0.8786	0.8935	0.8912	0.9383	0.8638	0.8660	0.9666	1.0000	0.9987	0.7634	0.6259	0.6177	0.9683	1.0000	1.0000
57	8p	PTK2B	rsPTK2B_8	rs2288617	inron 21	intron	27351026	0.4836	0.4646	0.4716	0.8425	0.6242	0.6149	0.7376	0.6372	0.6423	0.3815	0.1922	0.1809	0.5952	0.7729	0.7710	0.8786	0.8935	0.8912	0.9383	0.8638	0.8660	0.9666	1.0000	0.9987	0.7634	0.6259	0.6177	0.9683	1.0000	1.0000
58	8p	PTK2B	rsPTK2B_10	rs2241658	intron 25	intron	27353995	0.5312	0.5337	0.5411	0.8790	0.6854	0.6800	0.7819	0.7598	0.7591	0.4498	0.2430	0.2457	0.5175	0.7393	0.7449	0.8786	0.8935	0.8912	0.9383	0.8638	0.8660	0.9951	1.0000	0.9987	0.7634	0.6366	0.6287	0.9683	1.0000	1.0000
59	8p	PTK2B	rsPTK2B_11	rs2241659	intron 25	intron	27356005	0.2572	0.0959	0.0965	0.1302	0.0682	0.0652	0.3193	0.2471	0.2561	0.1266	0.0796	0.0708	0.2566	0.1853	0.1910	0.8786	0.8935	0.8912	0.6923	0.5213	0.5096	0.8733	0.8555	0.8720	0.6370	0.4018	0.3776	0.7850	0.9496	0.9218
60	8p	PTK2B	rsPTK2B_12	rs2241660	intron 25	intron	27356038	0.2666	0.1060	0.1076	0.1317	0.0700	0.0626	0.3111	0.2446	0.2487	0.1295	0.0816	0.0710	0.2455	0.1746	0.1835	0.8786	0.8935	0.8912	0.6923	0.5213	0.5096	0.8733	0.8555	0.8720	0.6370	0.4018	0.3776	0.7850	0.9496	0.9218
61	8p	PTK2B	rsPTK2B_15	rs4733064	intron 27	intron	27359043	0.5388	0.5202	0.5165	0.8888	0.6887	0.6820	0.7444	0.6548	0.6378	0.4258	0.2136	0.2059	0.5935	0.7776	0.7653	0.8786	0.8935	0.8912	0.9383	0.8638	0.8660	0.9666	1.0000	0.9987	0.7634	0.6259	0.6177	0.9683	1.0000	1.0000
62	8p	PTK2B	rsPTK2B_16	rs751019	exon 30	missense	27364502	0.6203	0.4353	0.4282	0.6114	0.3604	0.3564	0.5517	0.4234	0.4044	0.9955	1.0000	1.0000	0.0964	0.1060	0.0999	0.8786	0.8935	0.8912	0.9338	0.7480	0.7646	0.9666	0.9879	0.9478	0.9955	1.0000	1.0000	0.7850	0.8904	0.8851
63	8p	PTK2B	rsPTK2B_18	rs3735758	intron 31	intron	27366463	0.4517	0.5695	0.5768	0.8274	0.6189	0.6166	0.6372	0.9191	0.9200	0.4302	0.2133	0.2000	0.5270	0.6372	0.6329	0.8786	0.8947	0.8941	0.9383	0.8638	0.8660	0.9666	1.0000	1.0000	0.7634	0.6259	0.6177	0.9683	1.0000	1.0000
64	8p	PTK2B	rsPTK2B_19	rs1879184	exon 33	cds-synon	27367620	0.5086	0.5838	0.5937	0.8539	0.7190	0.7154	0.7089	0.7637	0.7589	0.5434	0.2844	0.2868	0.5601	0.8125	0.8118	0.8786	0.8947	0.8941	0.9383	0.8638	0.8660	0.9666	1.0000	0.9987	0.7870	0.6734	0.6883	0.9683	1.0000	1.0000
65	8p	PTK2B	rsPTK2B_20	rs10093964	intron 33	intron	27367957	0.1752	0.0664	0.0620	0.2352	0.0993	0.0976	0.1986	0.0956	0.0970	0.2759	0.1173	0.1165	0.1872	0.0789	0.0855	0.8786	0.8935	0.8912	0.7305	0.5213	0.5124	0.8733	0.6552	0.5820	0.7634	0.4479	0.4448	0.7850	0.8904	0.8851
66	8p	PTK2B	rsPTK2B_21	rs7007145	intron 34	intron	27368052	0.1761	0.0659	0.0665	0.2292	0.0851	0.0852	0.1717	0.1219	0.1207	0.2201	0.0820	0.0834	0.1957	0.1179	0.1159	0.8786	0.8935	0.8912	0.7305	0.5213	0.5096	0.8013	0.6826	0.6759	0.7634	0.4018	0.3776	0.7850	0.9003	0.8851
67	8p	PTK2B	rsPTK2B_23	rs1879182	exon 35	cds-synon	27371793	0.5042	0.5101	0.5114	0.8446	0.6455	0.6459	0.7350	0.6869	0.6816	0.4558	0.2228	0.2207	0.6313	0.8120	0.8056	0.8786	0.8935	0.8912	0.9383	0.8638	0.8660	0.9666	1.0000	0.9987	0.7634	0.6259	0.6287	0.9683	1.0000	1.0000
68	8p	PTK2B	rsPTK2B_24	rs1879181	exon 35	cds-synon	27371817	0.5039	0.5128	0.5148	0.8409	0.6518	0.6486	0.7341	0.6753	0.6842	0.4635	0.2310	0.2283	0.6317	0.8100	0.8119	0.8786	0.8935	0.8912	0.9383	0.8638	0.8660	0.9666	1.0000	0.9987	0.7634	0.6259	0.6287	0.9683	1.0000	1.0000
69	8p	NRG1	rsNRG1P_1	rs4281084	5' near gene	5' near gene	31614916	0.1938	0.9699	0.9699	0.2998	0.4979	0.4865	0.4339	0.5234	0.5029	0.1266	0.3545	0.3349	0.6591	0.5161	0.5037	0.8786	0.9816	0.9816	0.7782	0.8613	0.8617	0.8820	1.0000	0.9987	0.6370	0.7068	0.7060	0.9728	1.0000	1.0000
70	8p	NRG1	rsNRG1P_2	rs2200047	intron 1	intron	32196022	0.4702	0.8741	0.8780	0.9608	0.7852	0.7986	0.0461	0.2459	0.2393	0.8687	0.6338	0.6319	0.0925	0.2374	0.2406	0.8786	0.9732	0.9743	0.9608	0.9290	0.9448	0.3635	0.8555	0.8720	0.9438	0.8794	0.8721	0.7850	0.9496	0.9324
71	8p	NRG1	rsNRG1P_3	rs56228203	intron 1	intron	32196265	0.0525	0.0389	0.0365	0.1707	0.0613	0.0718	0.0184	0.3164	0.3213	0.0466	0.0154	0.0178	0.2255	0.6428	0.6499	0.8208	0.8935	0.8912	0.6923	0.5213	0.5096	0.3635	0.9165	0.9195	0.6370	0.4018	0.3776	0.7850	1.0000	1.0000
72	8p	NRG1	rsNRG1P_4	rs7832402	intron 1	intron	32196681	0.5713	0.9371	0.9389	0.9160	0.7198	0.7217	0.0447	0.3861	0.3790	0.7749	0.5118	0.5054	0.1416	0.3218	0.3206	0.8786	0.9732	0.9743	0.9383	0.8638	0.8660	0.3635	0.9879	0.9478	0.9041	0.8268	0.8230	0.7850	0.9672	0.9618
73	8p	NRG1	rsNRG1_6	rs7841677	intron 1	intron	32198397	0.1319	0.0471	0.0543	0.5069	0.2934	0.3076	0.0590	0.0586	0.0590	0.5236	0.5007	0.5095	0.0358	0.0444	0.0458	0.8208	0.8935	0.8912	0.8579	0.7157	0.7382	0.3980	0.5731	0.5820	0.7811	0.8247	0.8230	0.6014	0.8350	0.8148
74	8p	NRG1	rsSNRG1_2	rs4433107	intron 1	intron	32523368	0.8621	0.8816	0.8898	0.8058	0.5024	0.5105	0.7137	0.4827	0.4798	0.7648	0.4754	0.4853	0.7305	0.5431	0.5576	0.9660	0.9732	0.9743	0.9383	0.8613	0.8660	0.9666	1.0000	0.9987	0.9041	0.7987	0.8153	0.9773	1.0000	1.0000
75	8p	NRG1	rsNRG1P_5	rs3802159	intron 1	intron	32524243	0.3540	0.9375	0.9382	0.3331	0.2138	0.2219	0.3223	0.3132	0.3284	0.4549	0.2964	0.3027	0.3472	0.4930	0.4907	0.8786	0.9732	0.9743	0.7782	0.7157	0.7009	0.8733	0.9165	0.9195	0.7634	0.6734	0.7034	0.9124	1.0000	1.0000
76	8p	NRG1	rsNRG1_1	rs3924999	exon 2	missense	32572900	0.6171	0.7112	0.7007	0.7207	0.6212	0.6129	0.6298	1.0000	1.0000	0.6586	0.4462	0.4422	0.6601	0.9558	0.9551	0.8786	0.9008	0.8987	0.9383	0.8638	0.8660	0.9666	1.0000	1.0000	0.8257	0.7721	0.7581	0.9728	1.0000	1.0000
77	8p	NRG1	rsNRG1P_6	rs9642729	intron 3	intron	32590115	0.3828	0.7299	0.7275	0.5413	0.8609	0.8560	0.3443	0.2216	0.2165	0.4543	0.7372	0.7268	0.5454	0.3928	0.3850	0.8786	0.9016	0.8987	0.8579	0.9528	0.9468	0.8743	0.8555	0.8720	0.7634	0.8875	0.8861	0.9683	1.0000	1.0000
78	8p	NRG1	rsNRG1P_7	rs11989919	intron 3	intron	32622168	-	-	-	-	-	-	-	-	-	-	-	-	-	-	-	-	-	-	-	-	-	-	-	-	-	-	-	-	-	-
79	12q	DAO	rsDAO_5	rs2070585	intron 1	intron	107798175	0.7539	0.6707	0.6706	0.8380	0.8409	0.8380	0.2736	0.7351	0.7352	0.4357	0.4504	0.4357	0.6340	0.9180	0.9136	0.9092	0.8947	0.8941	0.9383	0.9528	0.9468	0.8733	1.0000	0.9987	0.7634	0.7721	0.7581	0.9683	1.0000	1.0000
80	12q	DAO	rsDAO_6	rs11114083	intron 1	intron	107801621	0.2918	0.1305	0.1305	0.1899	0.0880	0.0910	0.1693	0.5599	0.5672	0.0881	0.0357	0.0293	0.6301	0.6230	0.6223	0.8786	0.8935	0.8912	0.6923	0.5213	0.5096	0.8013	1.0000	0.9987	0.6370	0.4018	0.3776	0.9683	1.0000	1.0000
81	12q	DAO	rsDAO_7	rs2070586	intron 1	intron	107801849	0.4113	0.2086	0.1917	0.0491	0.0811	0.0700	0.2976	1.0000	1.0000	0.0511	0.0275	0.0252	0.9629	1.0000	1.0000	0.8786	0.8935	0.8912	0.6923	0.5213	0.5096	0.8733	1.0000	1.0000	0.6370	0.4018	0.3776	0.9773	1.0000	1.0000
82	12q	DAO	rsDAO_8	rs2070587	intron 1	intron	107801872	0.5906	0.3231	0.3197	0.3405	0.2207	0.2153	0.2376	1.0000	1.0000	0.1984	0.0749	0.0780	0.8349	0.8814	0.8783	0.8786	0.8935	0.8912	0.7782	0.7157	0.7009	0.8733	1.0000	1.0000	0.7454	0.4018	0.3776	0.9773	1.0000	1.0000
83	12q	DAO	rsDAO_13	rs55944529	intron 8	intron	107816559	0.0459	0.0313	0.0299	0.1978	0.1734	0.1700	0.0815	0.0392	0.0365	0.1669	0.0769	0.0812	0.2181	0.1551	0.1461	0.8208	0.8935	0.8912	0.6923	0.7157	0.7009	0.4890	0.5731	0.5820	0.6676	0.4018	0.3776	0.7850	0.9496	0.9218
84	13q	G72	rsG72_1	rs3916966	IGR	5' upstream	104908896	0.7421	0.4732	0.4679	0.8601	0.6210	0.6105	0.5738	0.3131	0.3182	0.9507	0.7644	0.7652	0.7360	0.4574	0.4527	0.9092	0.8935	0.8912	0.9383	0.8638	0.8660	0.9666	0.9165	0.9195	0.9808	0.8875	0.8861	0.9773	1.0000	1.0000
85	13q	G72	rsG72_E_1	rs45476401	exon 1	5' UTR	104916613	0.6380	0.6330	0.6404	0.6905	0.4736	0.4924	0.4109	0.8049	0.7906	0.5058	0.5493	0.5580	0.6941	0.9370	0.9376	0.8786	0.8947	0.8941	0.9383	0.8613	0.8617	0.8820	1.0000	1.0000	0.7811	0.8426	0.8511	0.9773	1.0000	1.0000
86	13q	G72	rsSG72_4	rs113692101	intron 1	intron	104916914	-	-	-	-	-	-	-	-	-	-	-	-	-	-	-	-	-	-	-	-	-	-	-	-	-	-	-	-	-	-
87	13q	G72	rsG72_E_2	rs2391191	exon2	missense	104917447	-	-	-	-	-	-	-	-	-	-	-	-	-	-	-	-	-	-	-	-	-	-	-	-	-	-	-	-	-	-
88	13q	G72	rsG72_6	rs778294	intron 3	intron	104940236	0.7328	0.5577	0.5614	0.6851	0.3828	0.392	0.7395	0.615	0.6263	0.8764	0.5917	0.6021	0.897	0.6494	0.6623	0.9092	0.8947	0.8941	0.93834	0.748	0.7646	0.9666	1	0.9987	0.9438	0.8704	0.87213	0.9773	1	1
89	13q	G72	rsG72_13	rs9558573	intron 4	intron	104941175	0.7527	0.6383	0.6401	0.8613	0.9651	0.9641	0.9456	0.8368	0.8371	0.6812	1	1	0.9773	0.8121	0.8082	0.9092	0.8947	0.8941	0.93834	0.9767	0.9777	1	1	1	0.8407	1	1	0.9773	1	1
90	22q	RASD2	rsRASD2_3	rs2272861	intron 1	intron	34267514	0.9679	0.821	0.8279	0.8527	0.604	0.609	0.9587	0.8141	0.8174	0.9591	0.8652	0.8703	0.8596	0.5944	0.5901	0.9775	0.9578	0.9659	0.93834	0.8638	0.866	1	1	1	0.9808	0.9439	0.94942	0.9773	1	1
91	22q	RASD2	rsRASD2_8	rs736212	exon 3	3' UTR	34278211	0.7129	0.4342	0.4306	0.5343	0.2594	0.2716	0.403	0.2503	0.2516	0.8051	0.5517	0.5472	0.2166	0.1009	0.1044	0.9092	0.8935	0.8912	0.85791	0.7157	0.7009	0.882	0.8555	0.872	0.9055	0.8426	0.8511	0.785	0.8904	0.8851
92	22q	RASD2	rsRASD2_9	rs736211	exon 3	3' UTR	34278239	0.6913	0.4088	0.4126	0.54	0.2589	0.2715	0.408	0.2432	0.2539	0.8085	0.5397	0.5512	0.2199	0.1049	0.1111	0.9092	0.8935	0.8912	0.85791	0.7157	0.7009	0.882	0.8555	0.872	0.9055	0.8426	0.8511	0.785	0.8904	0.8851
93	22q	RASD2	rsRASD2_10	rs6518958	exon 3	3' UTR	34279280	-	-	-	-	-	-	-	-	-	-	-	-	-	-	-	-	-	-	-	-	-	-	-	-	-	-	-	-	-	-
94	22q	CACNG2	rsCACNG2_3	rs2050141	intron 2	intron	35302102	0.0085	0.0018	0.0023	0.1774	0.0729	0.0697	0.0043	0.0013	0.0012	0.297	0.127	0.1347	0.0029	0.0006	0.0005	0.483	0.1512	0.1932	0.6923	0.5213	0.5096	0.1806	0.1092	0.1008	0.7634	0.4638	0.49195	0.112	0.0504	0.042
95	22q	CACNG2	rsCACNG2_16	rs2267360	intron 1	intron	35351483	0.8582	0.9384	0.9395	0.505	0.8621	0.8562	0.8303	0.7571	0.7609	0.3242	0.7713	0.7634	0.727	0.6017	0.6098	0.966	0.9732	0.9743	0.85791	0.9528	0.9468	1	1	0.9987	0.7634	0.8875	0.88614	0.9773	1	1
96	22q	CACNG2	rsCACNG2_15	rs2246071	intron 1	intron	35351741	0.9775	0.9022	0.9058	0.6418	0.9636	0.9659	0.7082	0.7959	0.8052	0.4049	0.9211	0.9205	0.5885	0.6671	0.674	0.9775	0.9732	0.9743	0.93834	0.9767	0.9777	0.9666	1	1	0.7634	0.9672	0.96653	0.9683	1	1
97	22q	CACNG2	rsCACNG2P_1	rs1883987	intron 1	intron	35383415	0.4614	0.3142	0.3077	0.4494	0.2354	0.2221	0.9311	0.944	0.9432	0.4176	0.1961	0.1997	0.8397	0.6043	0.6024	0.8786	0.8935	0.8912	0.83888	0.7157	0.7009	1	1	1	0.7634	0.6259	0.6177	0.9773	1	1
98	22q	CACNG2	rsCACNG2_20	rs2284006	intron 1	intron	35399975	0.5887	0.3846	0.383	0.3738	0.2132	0.2005	1	1	1	0.5027	0.2803	0.2715	0.8583	0.7069	0.6945	0.8786	0.8935	0.8912	0.78325	0.7157	0.7009	1	1	1	0.7811	0.6734	0.67076	0.9773	1	1
99	22q	CACNG2	rsCACNG2_18	rs916269	intron 1	intron	35400118	0.6044	0.3839	0.3879	0.3556	0.1934	0.1906	1	0.9468	0.9453	0.4587	0.2501	0.2383	0.8723	0.7532	0.7424	0.8786	0.8935	0.8912	0.77818	0.7157	0.7009	1	1	1	0.7634	0.6366	0.62873	0.9773	1	1
