# Supplementary material for: The DAO Gene Is Associated with Schizophrenia and Interacts with Other Genes in the Taiwan Han Chinese Population
Source: PLoS One. 2013 Mar 28;8(3):e60099. doi: 10.1371/journal.pone.0060099 (PMC3610748; doi:10.1371/journal.pone.0060099)
Supplement: Table S3 — Results of haplotype-based association tests. The results of haplotype-based association tests in an unstratified analysis of overall samples and a stratified analysis of four CPT strata, Zd’ ≥ −2.5, Zd’<−2.5, Zmd’ ≥ −2.5, and Zmd’<−2.5, are shown separately. In each sub-table, gene, LD block, haplotype, haplotype frequencies in case, control and combined groups, and the exact p-values and FDR-adjusted p-values of haplotype-based association tests are provided. (PDF) [file pone.0060099.s007.pdf]

Table S3. Results of haplotype-based association tests

overall

Gene	Block	Haplotype	Frequency			Haplotype-Trait Assoc.	
			Case	Control	Combined	Exact p	pFDR
DISC1	rsDISC1_35-rsDISC1_E_5	*C-C*	0.5935	0.6029	0.5971	0.6302	0.9810
		C-T	0.1747	0.1787	0.1762	0.7729	0.9810
		T-C	0.2318	0.2184	0.2267	0.4046	0.9810
LMBRD1	rsLMBRD1_8-rsLMBRD1_10-rsLMBRD1_3-rsLMBRD1_6-rsLMBRD1_11-rsLMBRD1_4	*A-C-C-T-T-T*	0.0683	0.0632	0.0663	0.5734	0.9810
		A-C-T-C-T-C	0.2800	0.2617	0.2730	0.2736	0.9810
		A-C-T-T-T-T	0.0532	0.0542	0.0536	0.8758	0.9810
		A-T-T-T-T-C	0.0476	0.0578	0.0515	0.2477	0.9810
		T-T-T-T-C-C	0.5464	0.5614	0.5522	0.4291	0.9810
DPYSL2	rsDPYSL_7-rsDPYSL_8-rsDPYSL_20-rsDPYSL_21-rsDPYSL_22-rsDPYSL_26	*A-C-C-G-C-T*	0.0923	0.0929	0.0933	0.9545	0.9810
		G-C-C-G-C-C	0.2273	0.2195	0.2234	0.6218	0.9810
		G-C-C-G-C-T	0.0538	0.0591	0.0560	0.5575	0.9810
		G-C-T-G-C-C	0.0538	0.0513	0.0530	0.7908	0.9810
		G-C-T-G-T-C	0.3999	0.4000	0.3989	0.9284	0.9810
		G-C-T-T-T-C	0.0554	0.0526	0.0545	0.7567	0.9810
		G-T-T-G-T-C	0.1035	0.1038	0.1046	0.8770	0.9810
TRIM35	rsSTRIM35_1-rsTRIM35_2-rsTRIM35_4-rsTRIM35_6	*A-G-T-G*	0.7603	0.7761	0.7663	0.3414	0.9810
		C-A-C-A	0.1999	0.1831	0.1934	0.2775	0.9810
		C-A-T-G	0.0258	0.0272	0.0264	0.8274	0.9810
PTK2B	rsPTK2B_28-rsPTK2B_29-rsPTK2B_30-rsSPTK2B_4-rsSPTK2B_5	*A-T-A-A-T*	0.2995	0.2780	0.2912	0.2172	0.9810
		A-T-A-G-T	0.1282	0.1408	0.1332	0.3222	0.9810
		G-G-G-G-G	0.5677	0.5785	0.5719	0.5480	0.9810
	rsSPTK2B_3-rsPTK2B_7-rsPTK2B_8-rsPTK2B_10-rsPTK2B_11-rsPTK2B_12-rsPTK2B_15-rsPTK2B_16-rsPTK2B_18-rsPTK2B_19-rsPTK2B_20-	*C-C-G-G-T-A-G-C-G-C-C-G-G-G*	0.0500	0.0476	0.0492	0.7701	0.9810
		T-C-G-G-C-G-G-A-G-C-T-A-G-G	0.2027	0.1768	0.1921	0.0930	0.8370
		T-C-G-G-C-G-G-C-G-C-C-G-G-G	0.0742	0.0740	0.0746	0.9435	0.9810
		T-C-G-G-T-A-G-C-G-C-C-G-G-G	0.1874	0.2079	0.1951	0.1721	0.9810
		T-T-A-A-T-A-A-A-A-T-C-G-A-A	0.4675	0.4736	0.4701	0.7552	0.9810
NRG1	rsNRG1P_2-rsNRG1P_3-rsNRG1P_4	*A-C-A*	0.2805	0.3177	0.2947	0.0374	0.4208
		A-T-A	0.2256	0.1877	0.2111	0.0169	0.2820
		G-T-G	0.4877	0.4883	0.4879	0.9695	0.9810
	rsSNRG1_2-rsNRG1P_5	*C-G*	0.1954	0.1905	0.1935	0.7429	0.9810
		T-C	0.6154	0.6147	0.6151	0.9688	0.9810
		T-G	0.1864	0.1895	0.1876	0.8385	0.9810
DAO	rsDAO_7-rsDAO_8-rsDAO_13	*A-G-C*	0.3622	0.3523	0.3585	0.5868	0.9810
		A-T-C	0.0124	0.0000	0.0077	0.0002	0.0090
		G-T-C	0.4809	0.4817	0.4811	0.9810	0.9810
		G-T-T	0.1338	0.1645	0.1457	0.0188	0.2820
G72	rsG72_1-rsG72_E_1	*A-C*	0.2839	0.2915	0.2868	0.6551	0.9810
		A-T	0.0862	0.0921	0.0885	0.6120	0.9810
		C-C	0.6299	0.6164	0.6247	0.4649	0.9810
RASD2	rsRASD2_8-rsRASD2_9	*C-G*	0.6204	0.6363	0.6265	0.4152	0.9810
		G-T	0.3791	0.3637	0.3732	0.4391	0.9810
CACNG2	rsCACNG2_16-rsCACNG2_15	*A-A*	0.5543	0.5541	0.5542	0.9700	0.9810
		G-G	0.4401	0.4350	0.4381	0.7893	0.9810
	rsCACNG2_20-rsCACNG2_18	*C-A*	0.8242	0.8375	0.8293	0.3455	0.9810
		T-T	0.1730	0.1606	0.1683	0.4056	0.9810

Gene	Block	Haplotype	Frequency			Haplotype-Trait Assoc.	
			Case	Control	Combined	Exact p	pFDR
		C-C	0.5980	0.6029	0.6007	0.8325	0.9082
DISC1	rsDISC1_35-rsDISC1_E_5	*C-T*	0.1700	0.1787	0.1748	0.6123	0.8644
		T-C	0.2320	0.2184	0.2244	0.4742	0.7764
		A-C-C-T-T-T	0.0541	0.0632	0.0591	0.4039	0.7764
	rsLMBRD1_8-rsLMBRD1_10-	*A-C-T-C-T-C*	0.2633	0.2617	0.2624	0.9505	0.9743
LMBRD1	rsLMBRD1_3-rsLMBRD1_6-	*A-C-T-T-T-T*	0.0473	0.0542	0.0511	0.4820	0.7764
	rsLMBRD1_11-rsLMBRD1_4	*A-T-T-T-T-C*	0.0450	0.0578	0.0521	0.2125	0.6940
		T-T-T-T-C-C	0.5847	0.5614	0.5717	0.3040	0.7296
		A-C-C-G-C-T	0.0841	0.0928	0.0893	0.5014	0.7764
		G-C-C-G-C-C	0.2245	0.2194	0.2215	0.7942	0.9077
	rsDPYSL_7-rsDPYSL_8-rsDPYSL_20-	*G-C-C-G-C-T*	0.0553	0.0591	0.0575	0.7283	0.8964
DPYSL2	rsDPYSL_21-rsDPYSL_22-	*G-C-T-G-C-C*	0.0587	0.0515	0.0548	0.4988	0.7764
	rsDPYSL_26	*G-C-T-G-T-C*	0.3991	0.3999	0.3989	0.9743	0.9743
		G-C-T-T-T-C	0.0574	0.0526	0.0549	0.6553	0.8765
		G-T-T-G-T-C	0.1095	0.1039	0.1068	0.7008	0.8852
	rsSTRIM35_1-rsTRIM35_2-	*A-G-T-G*	0.7410	0.7761	0.7604	0.0715	0.5347
TRIM35	rsTRIM35_4-rsTRIM35_6	*C-A-C-A*	0.2196	0.1831	0.1993	0.0470	0.5347
		C-A-T-G	0.0248	0.0272	0.0261	0.6756	0.8765
	rsPTK2B_28-rsPTK2B_29-	*A-T-A-A-T*	0.3153	0.2780	0.2945	0.0684	0.5347
	rsPTK2B_30-rsSPTK2B_4-	*A-T-A-G-T*	0.1261	0.1408	0.1344	0.3397	0.7521
	rsSPTK2B_5	*G-G-G-G-G*	0.5541	0.5785	0.5676	0.2387	0.6940
		A-A	0.3185	0.2829	0.2988	0.0729	0.5347
	rsPTK2B_1-rsPTK2B_2	*G-A*	0.2085	0.2126	0.2107	0.8234	0.9082
PTK2B		*G-G*	0.4672	0.4986	0.4846	0.1482	0.6112
	rsSPTK2B_3-rsPTK2B_7-rsPTK2B_8-	*C-C-G-G-T-A-G-C-G-C-C-G-G-G*	0.0518	0.0476	0.0495	0.6621	0.8765
	rsPTK2B_10-rsPTK2B_11-	*T-C-G-G-C-G-G-A-G-C-T-A-G-G*	0.2049	0.1768	0.1888	0.1107	0.5347
	rsPTK2B_12-rsPTK2B_15-	*T-C-G-G-C-G-G-C-G-C-C-G-G-G*	0.0808	0.0739	0.0775	0.5625	0.8433
	rsPTK2B_16-rsPTK2B_18-	*T-C-G-G-T-A-G-C-G-C-C-G-G-G*	0.1771	0.2079	0.1941	0.0844	0.5347
	rsPTK2B_19-rsPTK2B_20-	*T-T-A-A-T-A-A-A-A-T-C-G-A-A*	0.4696	0.4737	0.4720	0.8559	0.9130
		A-C-A	0.2782	0.3177	0.3001	0.0654	0.5347
	rsNRG1P_2-rsNRG1P_3-rsNRG1P_4	*A-T-A*	0.2185	0.1877	0.2014	0.1009	0.5347
NRG1		*G-T-G*	0.4944	0.4883	0.4910	0.7906	0.9077
		C-G	0.2055	0.1905	0.1972	0.4233	0.7764
	rsSNRG1_2-rsNRG1P_5	*T-C*	0.5896	0.6147	0.6035	0.2713	0.6940
		T-G	0.2021	0.1895	0.1951	0.4639	0.7764
		A-G-C	0.3701	0.3523	0.3603	0.4129	0.7764
DAO	rsDAO_7-rsDAO_8-rsDAO_13	*A-T-C*	0.0177	0.0000	0.0080	0.00007	0.00336
		G-T-C	0.4611	0.4817	0.4723	0.3447	0.7521
		G-T-T	0.1390	0.1645	0.1534	0.1114	0.5347
		A-C	0.3131	0.2915	0.3011	0.2747	0.6940
G72	rsG72_1-rsG72_E_1	*A-T*	0.0822	0.0921	0.0877	0.4502	0.7764
		C-C	0.6047	0.6164	0.6112	0.5798	0.8433
RASD2	rsRASD2_8-rsRASD2_9	*C-G*	0.6115	0.6363	0.6253	0.2691	0.6940
		G-T	0.3885	0.3637	0.3747	0.2691	0.6940
	rsCACNG2_16-rsCACNG2_15	*A-A*	0.5597	0.5541	0.5566	0.7877	0.9077
CACNG2		*G-G*	0.4336	0.4350	0.4344	0.9603	0.9743
	rsCACNG2_20-rsCACNG2_18	*C-A*	0.8142	0.8375	0.8272	0.1528	0.6112
		T-T	0.1824	0.1606	0.1703	0.1996	0.6940

Gene	Block	Haplotype	Frequency			Haplotype-Trait Assoc.	
			Case	Control	Combined	Exact p	pFDR
DISC1	rsDISC1_35-rsDISC1_E_5	*C-C*	0.5915	0.6029	0.5989	0.6652	0.9318
		C-T	0.1712	0.1787	0.1761	0.6887	0.9318
		T-C	0.2373	0.2184	0.2250	0.3868	0.8510
LMBRD1	rsLMBRD1_8-rsLMBRD1_10-rsLMBRD1_3-rsLMBRD1_6-rsLMBRD1_11-rsLMBRD1_4	*A-C-C-T-T-T*	0.0847	0.0632	0.0707	0.1164	0.7228
		A-C-T-C-T-C	0.2966	0.2617	0.2739	0.1106	0.7228
		A-C-T-T-T-T	0.0644	0.0542	0.0577	0.4545	0.8695
		A-T-T-T-T-C	0.0441	0.0578	0.0530	0.2159	0.7228
		T-T-T-T-C-C	0.5068	0.5614	0.5424	0.0280	0.4026
DPYSL2	rsDPYSL_7-rsDPYSL_8-rsDPYSL_20-rsDPYSL_21-rsDPYSL_22-rsDPYSL_26	*A-C-C-G-C-T*	0.0982	0.0928	0.0949	0.7189	0.9318
		G-C-C-G-C-C	0.2317	0.2194	0.2237	0.5551	0.9318
		G-C-C-G-C-T	0.0580	0.0591	0.0589	0.9389	0.9836
		G-C-T-G-C-C	0.0495	0.0514	0.0508	0.9040	0.9836
		G-C-T-G-T-C	0.4017	0.3999	0.3997	0.9525	0.9836
		G-C-T-T-T-C	0.0524	0.0526	0.0524	0.9806	0.9836
		G-T-T-G-T-C	0.0983	0.1038	0.1026	0.7273	0.9318
TRIM35	rsSTRIM35_1-rsTRIM35_2-rsTRIM35_4-rsTRIM35_6	*A-G-T-G*	0.7660	0.7761	0.7725	0.6624	0.9318
		C-A-C-A	0.1830	0.1831	0.1831	0.9836	0.9836
		C-A-T-G	0.0374	0.0272	0.0308	0.2803	0.7228
PTK2B	rsPTK2B_28-rsPTK2B_29-rsPTK2B_30-rsSPTK2B_4-rsSPTK2B_5	*A-T-A-A-T*	0.3034	0.2780	0.2864	0.2830	0.7228
		A-T-A-G-T	0.1271	0.1408	0.1365	0.4407	0.8695
		G-G-G-G-G	0.5644	0.5785	0.5736	0.5506	0.9318
	rsSPTK2B_3-rsPTK2B_7-rsPTK2B_8-rsPTK2B_10-rsPTK2B_11-rsPTK2B_12-rsPTK2B_15-rsPTK2B_16-rsPTK2B_18-rsPTK2B_19-rsPTK2B_20-	*C-G-G-C-G-G-A-G-C-T-A-G-G*	0.2100	0.1778	0.1881	0.1145	0.7228
		C-G-G-C-G-G-C-G-C-C-G-G-G	0.0695	0.0737	0.0728	0.7624	0.9318
		C-G-G-T-A-G-C-A-C-C-G-G-G	0.0102	0.0025	0.0053	0.0366	0.4026
		C-G-G-T-A-G-C-G-C-C-G-G-G	0.2272	0.2557	0.2462	0.1924	0.7228
		T-A-A-T-A-A-A-A-T-C-G-A-A	0.4695	0.4774	0.4746	0.7389	0.9318
NRG1	rsNRG1P_2-rsNRG1P_3-rsNRG1P_4	*A-C-A*	0.2931	0.3177	0.3092	0.2854	0.7228
		A-T-A	0.2391	0.1877	0.2055	0.0114	0.4026
		G-T-G	0.4627	0.4883	0.4794	0.3114	0.7228
	rsSNRG1_2-rsNRG1P_5	*C-G*	0.1793	0.1905	0.1866	0.5906	0.9318
		T-C	0.6437	0.6147	0.6247	0.2477	0.7228
		T-G	0.1750	0.1895	0.1845	0.4541	0.8695
DAO	rsDAO_7-rsDAO_8-rsDAO_13	*A-G-C*	0.3423	0.3523	0.3489	0.6925	0.9318
		A-T-C	0.0068	0.0000	0.0024	0.0002	0.0090
		G-T-C	0.5152	0.4817	0.4933	0.1870	0.7228
		G-T-T	0.1237	0.1645	0.1504	0.0252	0.4026
G72	rsG72_1-rsG72_E_1	*A-C*	0.2610	0.2915	0.2809	0.1867	0.7228
		A-T	0.0966	0.0921	0.0936	0.7596	0.9318
		C-C	0.6424	0.6164	0.6254	0.3121	0.7228
RASD2	rsRASD2_8-rsRASD2_9	*C-G*	0.6068	0.6363	0.6260	0.2515	0.7228
		G-T	0.3932	0.3637	0.3740	0.2515	0.7228
CACNG2	rsCACNG2_16-rsCACNG2_15	*A-A*	0.5491	0.5541	0.5524	0.8781	0.9836
		G-G	0.4458	0.4350	0.4387	0.6557	0.9318
	rsCACNG2_20-rsCACNG2_18	*C-A*	0.8390	0.8375	0.8380	0.9116	0.9836
		T-T	0.1593	0.1606	0.1602	0.9261	0.9836

Gene	Block	Haplotype	Frequency			Haplotype-Trait Assoc.	
			Case	Control	Combined	Exact p	pFDR
DISC1	rsDISC1_35-rsDISC1_E_5	*C-C*	0.6146	0.6029	0.6074	0.6040	0.8751
		C-T	0.1590	0.1787	0.1711	0.2928	0.8095
		T-C	0.2264	0.2184	0.2215	0.7086	0.9195
LMBRD1	rsLMBRD1_8-rsLMBRD1_10-rsLMBRD1_3-rsLMBRD1_6-rsLMBRD1_11-rsLMBRD1_4	*A-C-C-T-T-T*	0.0602	0.0632	0.0620	0.7896	0.9278
		A-C-T-C-T-C	0.2619	0.2617	0.2618	0.9908	0.9908
		A-C-T-T-T-T	0.0473	0.0542	0.0515	0.5148	0.8751
		A-T-T-T-T-C	0.0530	0.0578	0.0559	0.6375	0.8812
		T-T-T-T-C-C	0.5690	0.5614	0.5643	0.7434	0.9195
DPYSL2	rsDPYSL_7-rsDPYSL_8-rsDPYSL_20-rsDPYSL_21-rsDPYSL_22-rsDPYSL_26	*A-C-C-G-C-T*	0.0917	0.0928	0.0931	0.9338	0.9753
		G-C-C-G-C-C	0.2164	0.2194	0.2177	0.8818	0.9638
		G-C-C-G-C-T	0.0587	0.0591	0.0588	0.9850	0.9908
		G-C-T-G-C-C	0.0603	0.0514	0.0548	0.4416	0.8751
		G-C-T-G-T-C	0.3952	0.4000	0.3975	0.8537	0.9638
		G-C-T-T-T-C	0.0587	0.0526	0.0554	0.5826	0.8751
		G-T-T-G-T-C	0.1119	0.1039	0.1075	0.6004	0.8751
TRIM35	rsSTRIM35_1-rsTRIM35_2-rsTRIM35_4-rsTRIM35_6	*A-G-T-G*	0.7334	0.7761	0.7596	0.0409	0.3204
		C-A-C-A	0.2162	0.1831	0.1959	0.0854	0.4014
		C-A-T-G	0.0345	0.0272	0.0300	0.3563	0.8751
PTK2B	rsPTK2B_28-rsPTK2B_29-rsPTK2B_30-rsSPTK2B_4-rsSPTK2B_5	*A-T-A-A-T*	0.3223	0.2780	0.2950	0.0374	0.3204
		A-T-A-G-T	0.1390	0.1408	0.1402	0.9123	0.9745
		G-G-G-G-G	0.5358	0.5785	0.5620	0.0583	0.3914
	rsPTK2B_1-rsPTK2B_2	*A-A*	0.3209	0.2829	0.2976	0.0774	0.4014
		G-A	0.2235	0.2126	0.2168	0.5845	0.8751
		G-G	0.4498	0.4986	0.4798	0.0404	0.3204
	rsSPTK2B_3-rsPTK2B_7-rsPTK2B_8-rsPTK2B_10-rsPTK2B_11-rsPTK2B_12-rsPTK2B_15-rsPTK2B_16-rsPTK2B_18-	*C-G-G-C-G-G-A-G-C-T-A-G-G*	0.2063	0.1778	0.1883	0.1412	0.6033
		C-G-G-C-G-G-C-G-C-C-G-G-G	0.0802	0.0738	0.0770	0.6144	0.8751
		C-G-G-T-A-G-C-G-C-C-G-G-G	0.2464	0.2556	0.2520	0.6628	0.8900
		T-A-A-T-A-A-A-A-T-C-G-A-A	0.4513	0.4774	0.4673	0.2682	0.8095
NRG1	rsNRG1P_2-rsNRG1P_3-rsNRG1P_4	*A-C-A*	0.2636	0.3177	0.2968	0.0155	0.3204
		A-T-A	0.2249	0.1877	0.2021	0.0693	0.4014
		G-T-G	0.5014	0.4883	0.4933	0.5972	0.8751
	rsSNRG1_2-rsNRG1P_5	*C-G*	0.2053	0.1905	0.1962	0.4589	0.8751
		T-C	0.5892	0.6147	0.6048	0.2832	0.8095
		T-G	0.2002	0.1895	0.1936	0.5587	0.8751
DAO	rsDAO_7-rsDAO_8-rsDAO_13	*A-G-C*	0.3834	0.3523	0.3645	0.1789	0.7007
		A-T-C	0.0139	0.0000	0.0058	0.0001	0.0047
		G-T-C	0.4592	0.4817	0.4724	0.3417	0.8751
		G-T-T	0.1266	0.1645	0.1505	0.0272	0.3204
G72	rsG72_1-rsG72_E_1	*A-C*	0.3080	0.2915	0.2979	0.4255	0.8751
		A-T	0.0831	0.0921	0.0886	0.5126	0.8751
		C-C	0.6089	0.6164	0.6135	0.7261	0.9195
RASD2	rsRASD2_8-rsRASD2_9	*C-G*	0.6218	0.6363	0.6307	0.5639	0.8751
		G-T	0.3782	0.3637	0.3693	0.5639	0.8751
CACNG2	rsCACNG2_16-rsCACNG2_15	*A-A*	0.5602	0.5541	0.5565	0.7743	0.9278
		G-G	0.4312	0.4350	0.4335	0.8787	0.9638
	rsCACNG2_20-rsCACNG2_18	*C-A*	0.8166	0.8375	0.8295	0.2528	0.8095
		T-T	0.1819	0.1606	0.1689	0.2190	0.7918

Gene	Block	Haplotype	Frequency			Haplotype-Trait Assoc.	
			Case	Control	Combined	Exact p	pFDR
DISC1	rsDISC1_35-rsDISC1_E_5	*C-C*	0.5770	0.6029	0.5925	0.2861	0.8511
		C-T	0.1770	0.1787	0.1780	0.9151	0.9228
		T-C	0.2459	0.2184	0.2294	0.1766	0.7867
LMBRD1	rsLMBRD1_8-rsLMBRD1_10-rsLMBRD1_3-rsLMBRD1_6-rsLMBRD1_11-rsLMBRD1_4	*A-C-C-T-T-T*	0.0757	0.0632	0.0682	0.2920	0.8511
		A-C-T-C-T-C	0.2892	0.2617	0.2727	0.1788	0.7867
		A-C-T-T-T-T	0.0608	0.0542	0.0568	0.6060	0.9228
		A-T-T-T-T-C	0.0365	0.0578	0.0492	0.0434	0.5390
		T-T-T-T-C-C	0.5365	0.5614	0.5514	0.2854	0.8511
DPYSL2	rsSDPYSL2_5-rsDPYSL_7-rsDPYSL_8-rsDPYSL_20-rsDPYSL_21-rsDPYSL_22	*A-A-C-C-G-C*	0.0906	0.0944	0.0934	0.7754	0.9228
		A-G-C-C-G-C	0.2891	0.2766	0.2814	0.5544	0.9228
		A-G-C-T-G-T	0.3923	0.4000	0.3962	0.7583	0.9228
		A-G-C-T-T-T	0.0543	0.0521	0.0534	0.8547	0.9228
		A-G-T-T-G-T	0.0930	0.1077	0.1023	0.3095	0.8511
		G-G-C-T-G-C	0.0567	0.0497	0.0526	0.4966	0.9228
TRIM35	rsSTRIM35_1-rsTRIM35_2-rsTRIM35_4-rsTRIM35_6	*A-G-T-G*	0.7689	0.7761	0.7731	0.7327	0.9228
		C-A-C-A	0.1905	0.1831	0.1861	0.6793	0.9228
		C-A-T-G	0.0270	0.0272	0.0272	0.8801	0.9228
KPTK2B	rsPTK2B_28-rsPTK2B_29-rsPTK2B_30-rsSPTK2B_4-rsSPTK2B_5	*A-T-A-A-T*	0.2959	0.2780	0.2848	0.4060	0.9228
		A-T-A-G-T	0.1203	0.1408	0.1330	0.2147	0.8511
		G-G-G-G-G	0.5770	0.5785	0.5779	0.9228	0.9228
	rsSPTK2B_3-rsPTK2B_7-rsPTK2B_8-rsPTK2B_10-rsPTK2B_11-rsPTK2B_12-rsPTK2B_15-rsPTK2B_16-rsPTK2B_18-rsPTK2B_19-rsPTK2B_20-	*C-C-G-G-T-A-G-C-G-C-C-G-G-G*	0.0494	0.0476	0.0484	0.8596	0.9228
		T-C-G-G-C-G-G-A-G-C-T-A-G-G	0.2044	0.1768	0.1869	0.1452	0.7867
		T-C-G-G-C-G-G-C-G-C-C-G-G-G	0.0720	0.0739	0.0739	0.8926	0.9228
		T-C-G-G-T-A-G-C-G-C-C-G-G-G	0.1628	0.2079	0.1897	0.0146	0.3212
		T-T-A-A-T-A-A-A-A-T-C-G-A-A	0.4851	0.4737	0.4786	0.6285	0.9228
NRG1	rsNRG1P_2-rsNRG1P_3-rsNRG1P_4	*A-C-A*	0.3068	0.3177	0.3133	0.6180	0.9228
		A-T-A	0.2257	0.1877	0.2029	0.0490	0.5390
		G-T-G	0.4622	0.4883	0.4778	0.2570	0.8511
	rsSNRG1_2-rsNRG1P_5	*C-G*	0.1838	0.1905	0.1878	0.7236	0.9228
		T-C	0.6365	0.6147	0.6234	0.3489	0.9030
		T-G	0.1797	0.1895	0.1856	0.5842	0.9228
DAO	rsDAO_7-rsDAO_8-rsDAO_13	*A-G-C*	0.3390	0.3523	0.3471	0.5466	0.9228
		A-T-C	0.0109	0.0000	0.0043	0.0001	0.0044
		G-T-C	0.5013	0.4817	0.4895	0.3875	0.9228
		G-T-T	0.1378	0.1645	0.1539	0.1176	0.7392
G72	rsG72_1-rsG72_E_1	*A-C*	0.2757	0.2915	0.2852	0.4520	0.9228
		A-T	0.0905	0.0921	0.0915	0.8933	0.9228
		C-C	0.6338	0.6164	0.6234	0.4512	0.9228
RASD2	rsRASD2_8-rsRASD2_9	*C-G*	0.5986	0.6363	0.6212	0.1102	0.7392
		G-T	0.4014	0.3637	0.3788	0.1102	0.7392
CACNG2	rsCACNG2_16-rsCACNG2_15	*A-A*	0.5459	0.5541	0.5509	0.7383	0.9228
		G-G	0.4500	0.4350	0.4410	0.5088	0.9228
	rsCACNG2_20-rsCACNG2_18	*C-A*	0.8297	0.8375	0.8344	0.6672	0.9228
		T-T	0.1662	0.1606	0.1629	0.7853	0.9228
